# Spatial transcriptomics identifies differentiation, lipid metabolism, and retinoid pathway alterations in acne vulgaris

**DOI:** 10.1172/jci.insight.198021

**Published:** 2026-02-09

**Authors:** Joseph S. Durgin, Natalia A. Veniaminova, Thomas J. Huyge, Shih-Ying Tsai, Jennifer Fox, Yuli Cai, Mrinal K. Sarkar, Lam C. Tsoi, Johann E. Gudjonsson, Sunny Y. Wong

**Affiliations:** 1 Department of Dermatology,; 2 Department of Cell and Developmental Biology,; 3 Department of Computational Medicine and Bioinformatics, Michigan Medicine,; 4 Department of Biostatistics, School of Public Health, and; 5 Mary H Weiser Food Allergy Center, University of Michigan, Ann Arbor, Michigan, USA.

**Keywords:** Dermatology, Development, Skin, Therapeutics

## Abstract

Acne vulgaris is a common skin condition involving complex interactions among lipid-secreting sebaceous glands, keratinocytes, immune cells, and microbiota. While retinoids are effective for treating acne, disease pathogenesis remains poorly understood. In particular, it remains unclear how different subtypes of acne, including inflammatory (pustular) and noninflammatory (comedonal) lesions, vary in gene expression, signaling, and sebaceous gland involvement. Here, we performed spatial transcriptomics on healthy, nonlesional, comedonal, and pustular acne skin using a custom panel targeting sebaceous differentiation, lipid metabolism, and retinoid signaling pathways. We also designed a specialized segmentation pipeline to improve transcript assignment in the spatially complex sebaceous gland. Our analyses identified a PPARG^+^ transitional basal cell state in sebocytes and revealed that comedonal skin upregulates sebogenesis genes, whereas pustular skin downregulates sebogenesis. Both lesion types exhibited increased AP-1 transcription factors and elevated FABP5, a chaperone that blunts retinoic acid receptor signaling. Finally, we demonstrated that an AP-1 inhibitor, T-5224, downregulates FABP5 in human keratinocytes and reduces pustule formation in a mouse model of high-fat diet–induced folliculitis. Altogether, these findings indicate that altered lipogenesis, retinoid signaling, and keratinocyte differentiation are key features of acne, and nominate AP-1 and FABP5 as potential therapeutic targets.

## Abstract 2

Our study characterizes gene expression changes that occur during acne pathogenesis. We also identify a novel therapeutic that reduces acne-like pustule formation in a mouse model of high fat diet-induced folliculitis.

## Introduction

Acne vulgaris is among the most prevalent skin diseases worldwide, affecting approximately 85% of adolescents and 9.4% of the global population (1, 2). In the United States alone, acne accounted for an estimated $846 million in medical costs and $398 million in productivity losses in 2013 (1, 3). Furthermore, patients experience stigmatization, increased risk of depression, and negative psychological well-being comparable with those with other chronic diseases such as asthma, diabetes, arthritis, and epilepsy (1, 4, 5).

Acne pathogenesis is characterized by altered sebaceous gland function, follicular hyperkeratinization, microbial dysbiosis, hormonal influences, altered immune responses, and skin barrier impairment (6–12). Acne risk and severity are also thought to be increased by diets rich in carbohydrates, saturated fats, and dairy products (13). Comedonal lesions are characterized by altered keratinocyte (KC) differentiation and hyperkeratinization in the upper hair follicle domain known as the infundibulum (14, 15). Notably, acne lesions are typically localized to skin with a high density of sebaceous glands (16, 17), which secrete lipid-containing sebum that flows onto the surface of the skin (18–20). In addition, acne onset often coincides with increased sebaceous gland activity, secondary to androgens and growth factors during puberty (9). *Cutibacterium acnes*, the microorganism most frequently associated with acne pathogenesis, colonizes sebaceous glands and metabolizes sebaceous lipids (6). Overall, the multifaceted association of acne with sebaceous gland development and function suggests a necessary, if not instigating, role for these glands in disease.

For severe, disfiguring acne, the most effective treatment is oral isotretinoin (13-*cis*-retinoic acid), a first-generation retinoid with antiproliferative and proapoptotic effects on sebocytes (21, 22). Mechanistically, isotretinoin is thought to undergo intracellular isomerization to all-trans retinoic acid (ATRA) (13). Subsequently, chaperone proteins such as FABP5 and CRABP2 compete to bind to ATRA and deliver it to different nuclear receptors (13). Critically, the ratio of FABP5/CRABP2 can lead to starkly divergent outcomes. In cells with high FABP5, ATRA activates PPAR-β/δ, leading to increased growth and survival. In cells with high CRABP2, on the other hand, ATRA activates retinoic acid receptors (RARs), which induce differentiation and apoptosis (23). In the most inflammatory forms of acne, patients can experience severe paradoxical flares in response to isotretinoin (24), and isotretinoin use can cause birth defects, underscoring the need to better understand how retinoic acid signaling is dysregulated in acne and to identify alternative therapeutic approaches.

Prior gene expression studies in acne have focused primarily on inflammatory lesions (papules, pustules, and nodules) (25–28). Analyses using microarrays or bulk RNA-Seq have identified upregulation of numerous mRNA transcripts in acne compared with healthy skin, including genes encoding matrix metalloproteinases (MMP1, MMP3, and MMP9), cytokines (TNF-α, IL-1β, IL-8, and IL-10), antimicrobial peptides (DEFB4), and immune cell markers (CD28, CD163, GZMB) (25, 27). Profiling studies in acne have also identified increased cell-cell communication involving CXCL8, a potent chemoattractant that may in part explain the dense neutrophilic infiltrate in inflammatory acne (28). However, the absence of noninflammatory comedones in most studies limits analysis of the transition from the earliest event in acne pathogenesis — infundibular hyperkeratinization — to pustular acne.

Over the past few years, single-cell RNA-Seq (scRNA-Seq) studies have identified disease-specific signatures in hair follicle KC and immune cell compartments in acne (14, 26, 28, 29). Despite their central importance in acne pathogenesis, however, sebaceous glands have not been a focus of scRNA-Seq profiling of acne skin thus far (30). While scRNA-Seq is, in theory, well poised to capture this heterogeneity, differentiated sebocytes are large, lipid-filled, and fragile, which frequently leads to their loss during tissue processing; consequently, these cells are often underrepresented by scRNA-Seq profiling (26, 30). Spatial transcriptomics, which can detect mRNA transcripts even in fragile cells in situ, enables the sebaceous gland to be profiled at single-cell resolution (31–33).

Here, we analyze healthy, nonlesional, and acne lesional skin using spatialomics. We focus on the sebaceous gland and follicular KC compartments, utilizing a targeted panel encompassing genes critical for differentiation, lipid metabolism, and signaling. To better study gene expression in the complex microanatomy of the sebaceous gland, we also develop and validate a custom *Keratin 5*–directed (*KRT5*-directed) cell segmentation approach. By comparing acne disease states with healthy skin, we elucidate comedonal- and pustule-specific alterations, and we nominate AP-1 and FABP5 as targets for therapy.

## Results

### KRT5-directed segmentation defines basal sebocyte progenitors.

**Figure 1:**
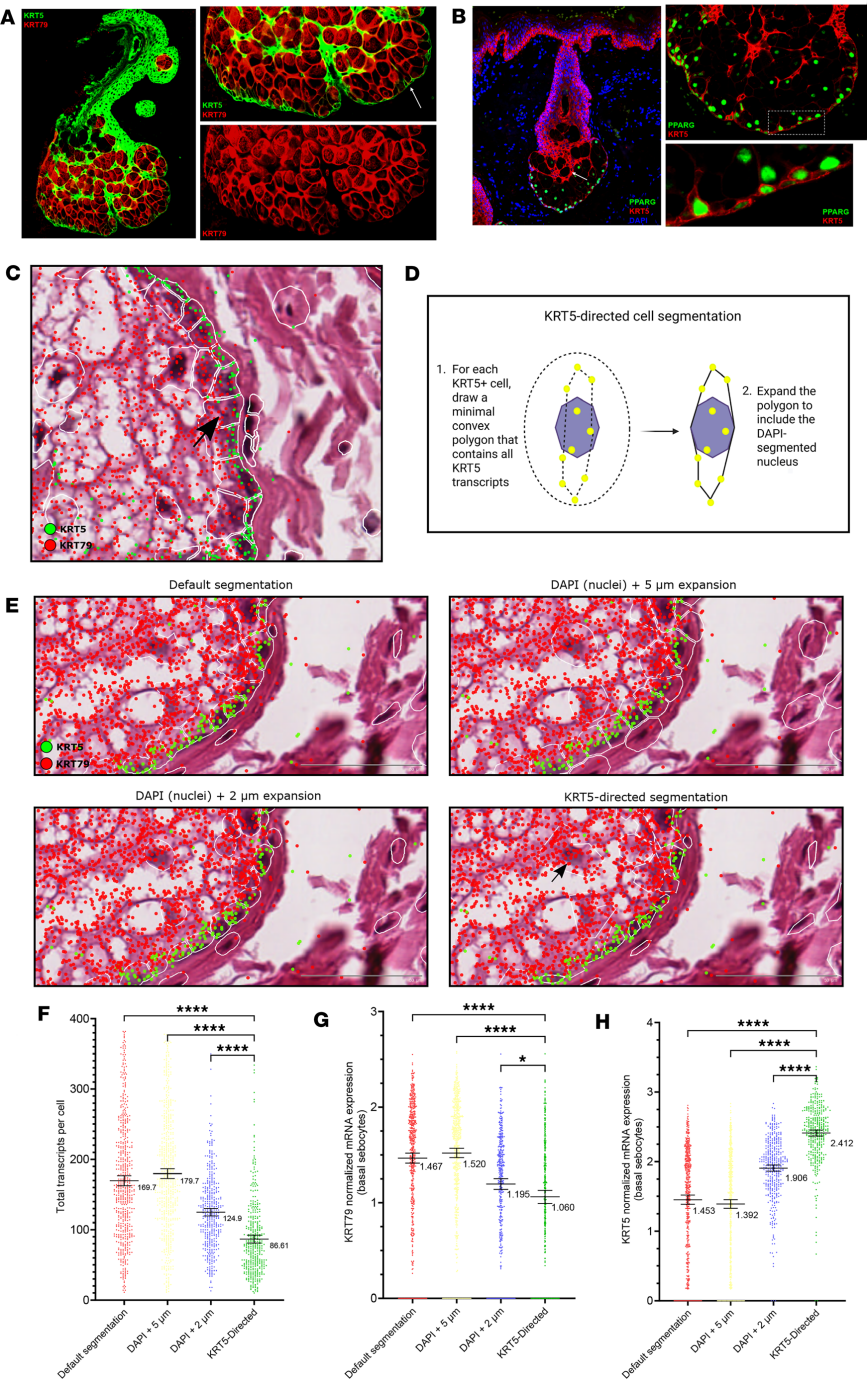
Sebaceous gland microanatomy and evaluation of different segmentation approaches. (**A**) Normal sebaceous gland stained for KRT5 (green) and KRT79 (red). The white arrow indicates the thin KRT5^+^ basal cell layer at the periphery. (**B**) KRT5 (red) and PPARG (green) in healthy skin. A subset of peripheral KRT5^+^ basal cells coexpresses PPARG, similar to in mice (34). KRT5^+^ septations (arrow) usually do not express PPARG. (**C**) Cell boundaries (white lines) drawn by default Xenium segmentation with multimodal segmentation fail to capture the slender contours of peripheral sebaceous gland basal cells (arrow). (**D**) Depiction of our custom segmentation approach, using *KRT5* transcript coordinates (yellow dots) to refine cell boundaries (oval dotted line represents nuclear expansion-based cell contours). (**E**) Comparison of 4 segmentation methods (default, 2 μm nuclear expansion, 5 μm nuclear expansion, and *KRT5*-directed) in a representative healthy sebaceous gland. The black arrow points to a differentiated sebocyte (*KRT5*^–^), where multimodal information is preserved using default parameters. (**F**–**H**) Comparison of total transcripts per cell, *KRT79* normalized expression, and *KRT5* normalized expression across segmentation methods in sebaceous gland basal cells from a representative healthy sample. Statistical significance was calculated using a 1-way ANOVA with post hoc *t* test and Tukey’s correction for multiple comparisons (*P < 0.05; ***P < 0.001; ****P < 0.0001). Data are shown as mean ± 95% CI. Scale bars: 50 μm.

Human sebaceous glands are multilobulated appendages that secrete an oily substance known as sebum through an excretory duct connected to the hair follicle infundibulum. At the periphery of each lobule is a thin monolayer of mitotically active, KRT5^+^ basal stem cells that maintain the gland (Figure 1A and Supplemental Figure 1A; supplemental material available online with this article; https://doi.org/10.1172/jci.insight.198021DS1). A subset of KRT5^+^ basal cells at the bottom of the gland also coexpress PPARG (Figure 1B), which, in mice, are thought to represent transitional cells at early stages of differentiation (34). Unique to human sebaceous glands, we also frequently observed internally located KRT5^+^ basal cell “septations,” which are usually PPARG^–^ (Figure 1B). Finally, we noted that differentiated sebocytes express lower levels of KRT5 and high levels of a different keratin, KRT79 (Figure 1A), consistent with mouse sebaceous glands (34, 35). Together, these markers provide key molecular landmarks to understand sebocyte differentiation.

The multimodal Xenium segmentation kit uses a cocktail of antibodies against membrane (ATP1A1, E-Cadherin, CD45) and cytoplasmic (18S ribosomal RNA, α-SMA, vimentin) protein markers, along with a deep learning algorithm, to infer cell boundaries for transcript assignment. By visual inspection, we noticed that this segmentation method overestimated the slender contours of sebaceous gland basal cells, incorrectly extending their boundaries into the lipid-filled spaces occupied by adjacent differentiated sebocytes (Figure 1C). This caused transcripts encoding differentiated sebocyte markers such as *KRT79* and *AWAT2* to be erroneously assigned to the basal cell compartment (Figure 1C and Supplemental Figure 2A).

Given the basal-enriched expression of KRT5 in the sebaceous gland, we therefore developed a custom routine in which *KRT5* transcript coordinates are used to refine the cell boundaries generated by the default segmentation approach (Figure 1D). For cells that do not express *KRT5*, their original boundaries are retained according to the standard multimodal segmentation approach.

We compared our *KRT5*-driven combined approach against multimodal segmentation alone and also against traditional approaches using 2 μm and 5 μm nuclei expansion–based segmentation (36). Compared with other methods, our *KRT5*-driven approach yielded slender basal cell contours better resembling those seen by H&E and IHC staining (Figure 1, A, B, and E). As expected, the *KRT5*-driven method yielded smaller cell sizes, fewer transcripts per cell, decreased *KRT79* and *AWAT2* transcript assignment in basal progenitors, and increased normalized *KRT5* expression in basal cells (Figure 1, F–H, and Supplemental Figure 2A). Since our *KRT5*-driven approach incorporates the standard segmentation kit cell boundaries as input, this method retains the information derived from the cocktail of membrane, cytoplasmic, and interior stains for all cells (Figure 1E, arrow).

### Spatial transcriptomics identifies gene expression alterations in acne.

**Figure 2:**
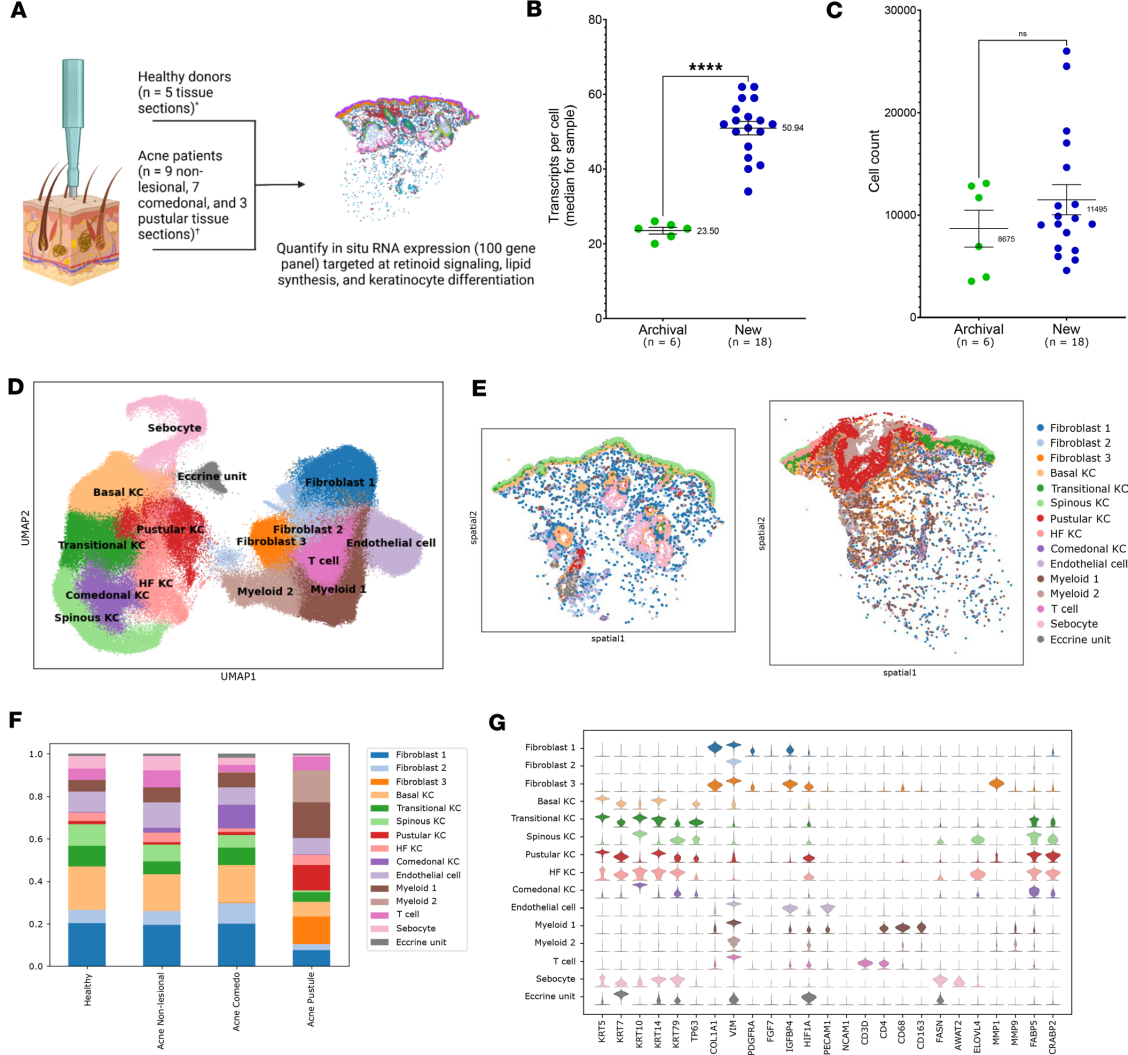
Global cell clustering and marker gene expression in healthy and acne skin. (**A**) Schematic of study design and samples submitted for spatial transcriptomics analysis. (**B** and **C**) Scatter plots of total transcripts per cell and cell counts per sample, for archival and new samples, after quality control. (**D**) UMAP clustering of cells pooled from all samples. (**E**) Spatial plot of cells labeled by cluster, in representative healthy and pustule samples. (**F**) Proportion of cell cluster types found across all samples. (**G**) Violin plots depicting marker genes and DEGs for each cluster. Statistical significance was determined using a *t* test (****P < 0.0001). Data are shown as mean ± 95% CI.

Utilizing our combined segmentation approach, we performed spatial transcriptomics analysis on 24 tissue sections (5 healthy skin, 9 nonlesional skin from acne patients, 7 comedonal, 3 pustular) from 11 donors (Figure 2A and Supplemental Table 1 for patient characteristics), using a custom 100-gene panel enriched for genes associated with sebaceous differentiation, lipid metabolism, and retinoid signaling (Supplemental Table 2). Of these samples, 6 were archival and collected > 10 years ago, while the remaining 18 samples were recently collected over the past 12 months. The median number of transcripts per cell averaged 23.5 and 50.9 in the archival and recent specimens, respectively (Figure 2B). Nonetheless, the number of high-quality cells meeting thresholds for analysis (containing ≥ 10 transcripts with quality values ≥ 20) was comparable between archival and recent samples (Figure 2C) (37).

After merging all healthy and acne specimens into a single gene expression matrix, we identified 15 cell type–specific clusters using uniform manifold approximation and projection (UMAP) clustering (Figure 2, D–F). This included 4 KC subpopulations in healthy skin: Basal KC, Transitional KC, Spinous KC, and Hair Follicle (HF) KC. We also identified 2 disease-specific KC subpopulations: Pustular KC and Comedonal KC. Non-KC cell types enriched in pustular skin included certain myeloid and fibroblast subpopulations (Figure 2, E and F).

Based on analysis of differentially expressed genes (DEGs), the Comedonal KC subpopulation was characterized by higher *KRT10* expression and reduced *KRT14* and *KRT79* compared with normal HF KC (Figure 2G). *KRT79* is normally highly expressed in differentiated cells lining the hair follicle infundibulum, in addition to the sebaceous gland (15). The Pustular KC cluster represented a less-differentiated, more basal-like KC population (*KRT5*^+^*KRT14*^+^) that notably had increased *HIF1A* and *MMP1* but low *KRT10* (Figure 2G). Finally, the sebaceous gland initially emerged as a single cluster expressing the canonical marker genes *FASN* and *AWAT2*, in addition to *KRT14* and *KRT79* (Figure 2G).

### Dysregulated lipogenesis in acne-associated sebaceous glands.

**Figure 3:**
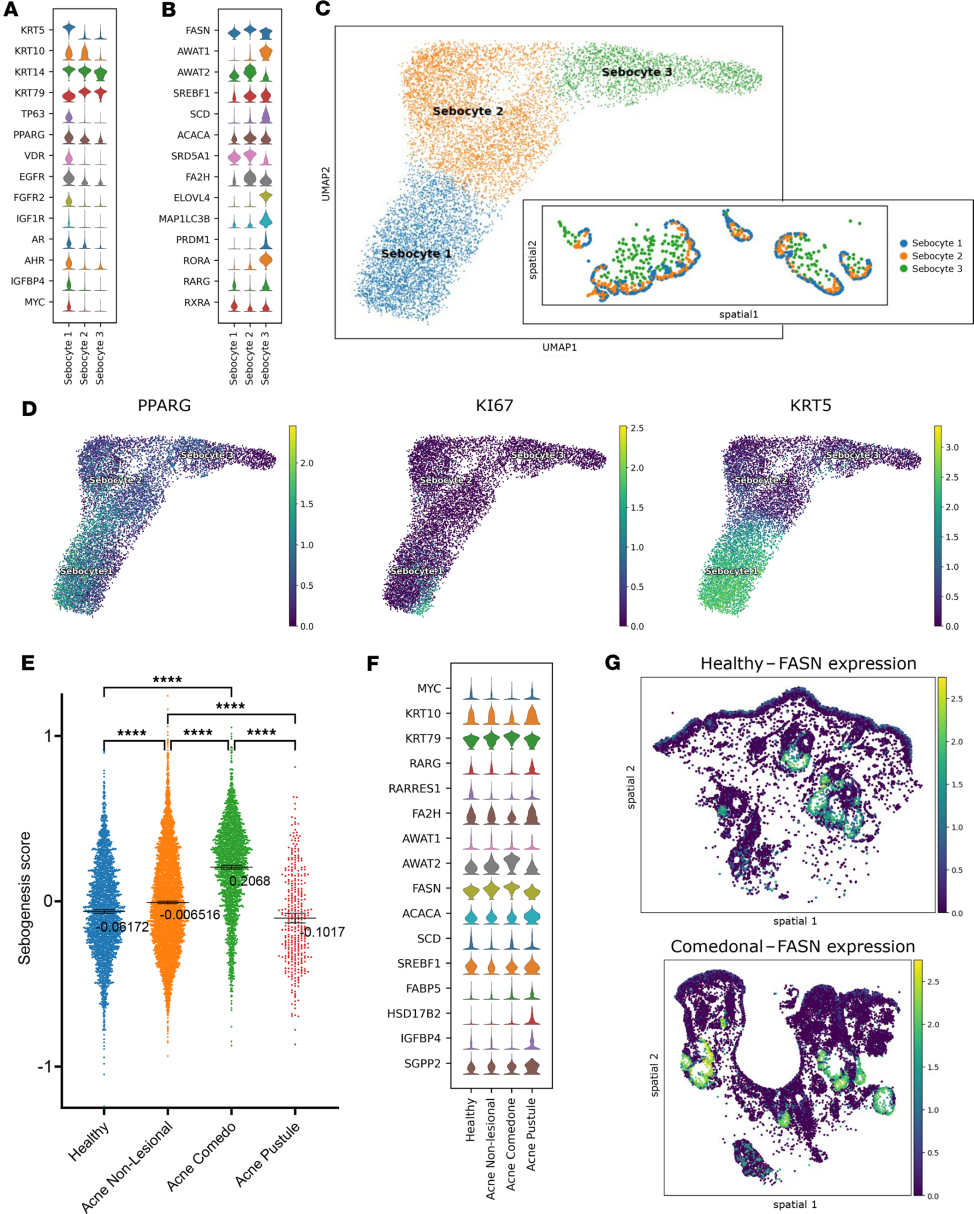
Sebaceous gland spatial transcriptomics analysis. (**A**) Subclustering on pooled sebocytes from all specimens, revealing 3 subpopulations (Sebocyte 1–3). Sebocyte 1 (basal) highly expresses *KRT5*, *PPARG*, and *TP63*. Sebocyte 2 and 3 (differentiated) lose *KRT5* and gain *KRT79*. (**B**) Expression of lipid and retinoid metabolism genes across Sebocyte clusters. (**C**) UMAP plot of all Sebocyte clusters. The inset shows the spatial coordinates of cells, labeled by cluster, in a representative healthy sample. (**D**) UMAP plot of Sebocyte subclusters labeled by expression of *PPARG*, *MKI67* (Ki-67), and *KRT5*. (**E**) Sebogenesis score across different skin conditions, calculated in sebocytes based on composite lipogenic gene expression (*FASN*, *AWAT1*, *AWAT2*, *ACACA*, *SREBF1*). (**F**) Notable DEGs among all pooled sebocytes, separated by disease state. (**G**) Spatial distribution of normalized *FASN* expression in representative healthy and comedonal samples. Statistical significance was determined using a 1-way ANOVA with post hoc *t* test and Tukey’s correction for multiple comparisons (****P < 0.0001). Data are shown as mean ± 95% CI.

To better delineate distinct stages of sebocyte differentiation, we performed subclustering on the pooled sebocytes from all specimens, which identified 3 subpopulations (Figure 3, A–C). Sebocyte 1 spatially corresponds to the basal KRT5^+^ population (Figure 3C, inset). This basal population highly expresses *PPARG*, *EGFR*, *FGFR2*, *IGF1R*, *VDR*, and *AR* compared with more differentiated Sebocytes 2 and 3, suggesting increased growth factor and hormonal sensitivity in these progenitor cells. These basal sebocytes also express *TP63*, along with the retinoid receptors *RARG* and *RXRA* (Figure 3B). Notably, proliferative basal cells (*MKI67*^hi^) were *KRT5*^+^*PPARG*^–^, lending support to the model that KRT5^+^PPARG^+^ basal sebocytes are less proliferative and, thus, represent transitional cells poised to differentiate (Figure 3D and Supplemental Figure 1A) (34). Of note, the small fraction of *MKI67*^+^ basal sebocytes observed by spatial transcriptomics (Figure 3D) is consistent with the relatively sparse Ki67 protein staining seen by IHC in the basal layer of sebaceous glands (Supplemental Figure 1A)

Compared with basal cells, the Sebocyte 2 population expresses higher levels of lipogenesis enzymes including *FASN*, *AWAT2*, *ACACA*, and *FA2H* (Figure 3B). Sebocyte 2 also expresses the highest level of *SRD5A1*, encoding 5α-reductase, suggesting that these early-differentiated sebocytes may be critical for converting testosterone to the more potent dihydrotestosterone (DHT). Sebocyte 3, the most differentiated and centrally located sebocytes, are characterized by high expression of *AWAT1*, *SCD*, *ELOVL4*, *RORA*, and *MAP1LC3B*, which encodes a regulator of autophagy (Figure 3B). Prior studies have shown that autophagy is critical for terminal sebocyte differentiation (38).

Next, we compared gene expression across disease states (healthy, nonlesional, comedo, pustule) specifically in the sebaceous gland compartment. As a global assessment of gland function, we scored cells based on the composite expression of 5 genes (*FASN*, *AWAT1*, *AWAT2*, *ACACA*, *SREBF1*) critical for sebaceous lipogenesis. As expected, the highest sebogenesis score was observed in comedone-associated sebaceous glands (Figure 3, E–G). Unexpectedly, pustule-associated sebaceous glands exhibited lower sebogenesis scores than did glands found in healthy or nonlesional skin (Figure 3E), raising the possibility that the acutely inflamed microenvironment of the pustule reduces sebaceous gland function. Pustule tissue also had proportionately fewer sebocytes (Figure 2F). Finally, sebaceous glands in comedonal samples showed evidence of decreased retinoic acid signaling, with lower *RARG* and *RARRES1* expression (Figure 3F). Decreased expression of retinoid pathway components and increased *FASN* in comedones was also observed in paired acne versus nonlesional samples collected from a single patient (Supplemental Figure 3A).

### Altered differentiation and retinoid signaling in acne KCs.

**Figure 4:**
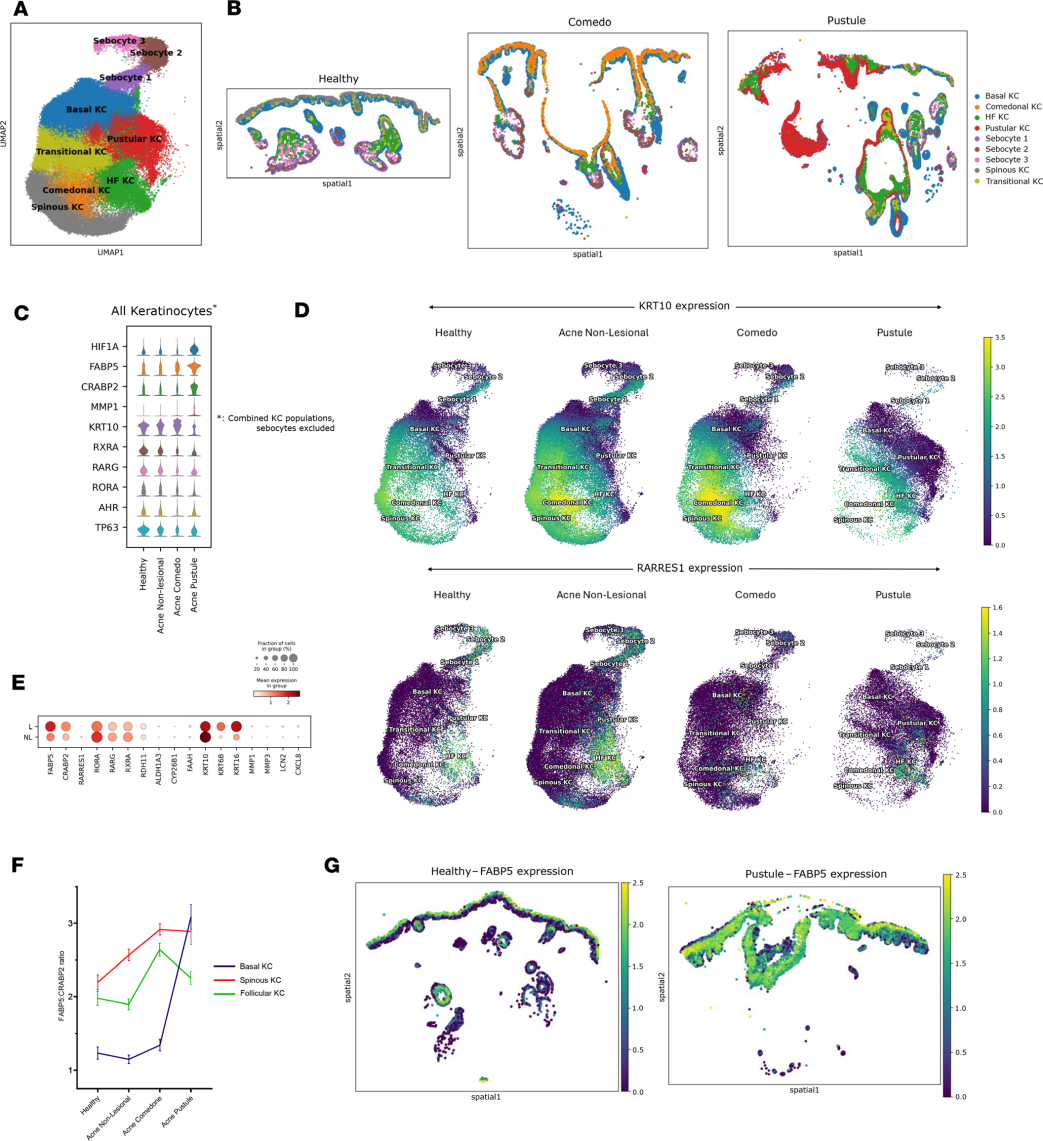
Analysis of pooled keratinocytes. (**A**) UMAP plot of pooled keratinocyte clusters and sebocyte subclusters across all samples. (**B**) Spatial cell plots for representative healthy, comedo, and pustule samples. (**C**) Expression of select DEGs in pooled keratinocytes across disease states (without sebocytes). (**D**) Normalized *KRT10* or *RARRES1* expression in UMAPs across disease states. (**E**) Selected DEGs from an scRNA-Seq dataset by Do et al., comparing inflammatory acne lesional (L) versus nonlesional (NL) gene expression in keratinocytes (26). (**F**) *FABP5*:*CRABP2* gene expression ratio in basal, spinous, and follicular keratinocytes across disease states in our spatial transcriptomic data. Data are shown as mean ± 95% CI. (**G**) Normalized *FABP5* expression in healthy and pustular samples.

We next analyzed all nonsebocyte KCs collectively to control for disease-associated shifts in cluster identity (Figure 4, A–D). We observed that KCs in comedonal lesions display increased *KRT10* and *FABP5* expression and decreased *CRABP2*, *TP63*, and *AHR*, relative to healthy or acne nonlesional skin (Figure 4, B and C). Pustular KCs exhibit higher expression of *FABP5* and *HIF1A* compared with healthy and acne nonlesional skin, and exhibit low *KRT10*. Notably, KCs in both comedones and pustules have reduced expression of genes encoding retinoid receptors (*RXRA*, *RARG*, *RORA*) (Figure 4C). Concordantly, our analysis of an independent scRNA-Seq dataset of inflammatory acne lesions paired with nonlesional controls (26) also revealed reduced *KRT10*, reduced expression of retinoid receptors, and increased *FABP5* in lesional KCs (Figure 4E).

As noted above, the HF KC cluster was primarily found in healthy skin, where these cells express markers of upper hair follicle identity, including *KRT79* and *GATA6*. Separating the UMAP plots by disease state, we observed that this HF KC cluster is reduced in comedonal lesions, being replaced by the *KRT10*^hi^ Comedonal KC subpopulation (Figure 2F and Figure 4, B and D). This is consistent with the fact that acne arises from the upper hair follicle and perturbs differentiation in this domain. In pustules, the HF KC cluster is replaced instead by the Pustular KC subpopulation, which is characterized by lower *KRT10* expression (Figure 2F and Figure 4, B and D). *KRT79* is reduced in comedones (Supplemental Figure 4A), consistent with our previous findings (15), while *GATA6* is lost in both comedones and pustules (Supplemental Figure 4B) (14). These findings suggest that acne is associated with altered hair follicle differentiation, with comedo-associated follicles adopting an epidermal hyperkeratinizing phenotype (*KRT10*^hi^), and pustule-associated follicles taking on a more dedifferentiated, hyperplastic basal-like state (*KRT5*^hi^*KRT14*^hi^*KRT10^lo^*). Finally, *RARRES1*, a target gene of retinoic acid signaling, is also reduced in both comedones and pustules (Figure 4D and Supplemental Figure 4C). These alterations in *KRT10*, *FABP5*, and retinoid pathway components were mirrored when KC populations from paired lesional and nonlesional samples were compared from the same individual (Supplemental Figure 3B).

Previous studies have shown that the balance between FABP5 and CRABP2 is critical for determining whether retinoic acid activates PPAR-β/δ or RARs, respectively, with opposing effects on cell growth, differentiation, and survival (23, 39). Therefore, we evaluated the ratio of *FABP5* and *CRABP2* expression for all nonsebocyte KCs across disease states (Figure 4F). We found that all lesional KC subpopulations generally exhibit increased *FABP5*/*CRABP2* ratios (Figure 4F). Notably, basal KCs from pustules display far higher *FABP5*/*CRABP2* ratios than did basal KCs from any other condition (Figure 4, F and G). Altogether, these findings suggest that elevated *FABP5*/*CRABP2* ratios in acne comedones and pustules led to preferential activation of PPAR-β/δ in response to retinoic acid, resulting in hyperproliferation.

### FABP5 and KRT10 are altered in acne.

**Figure 5:**
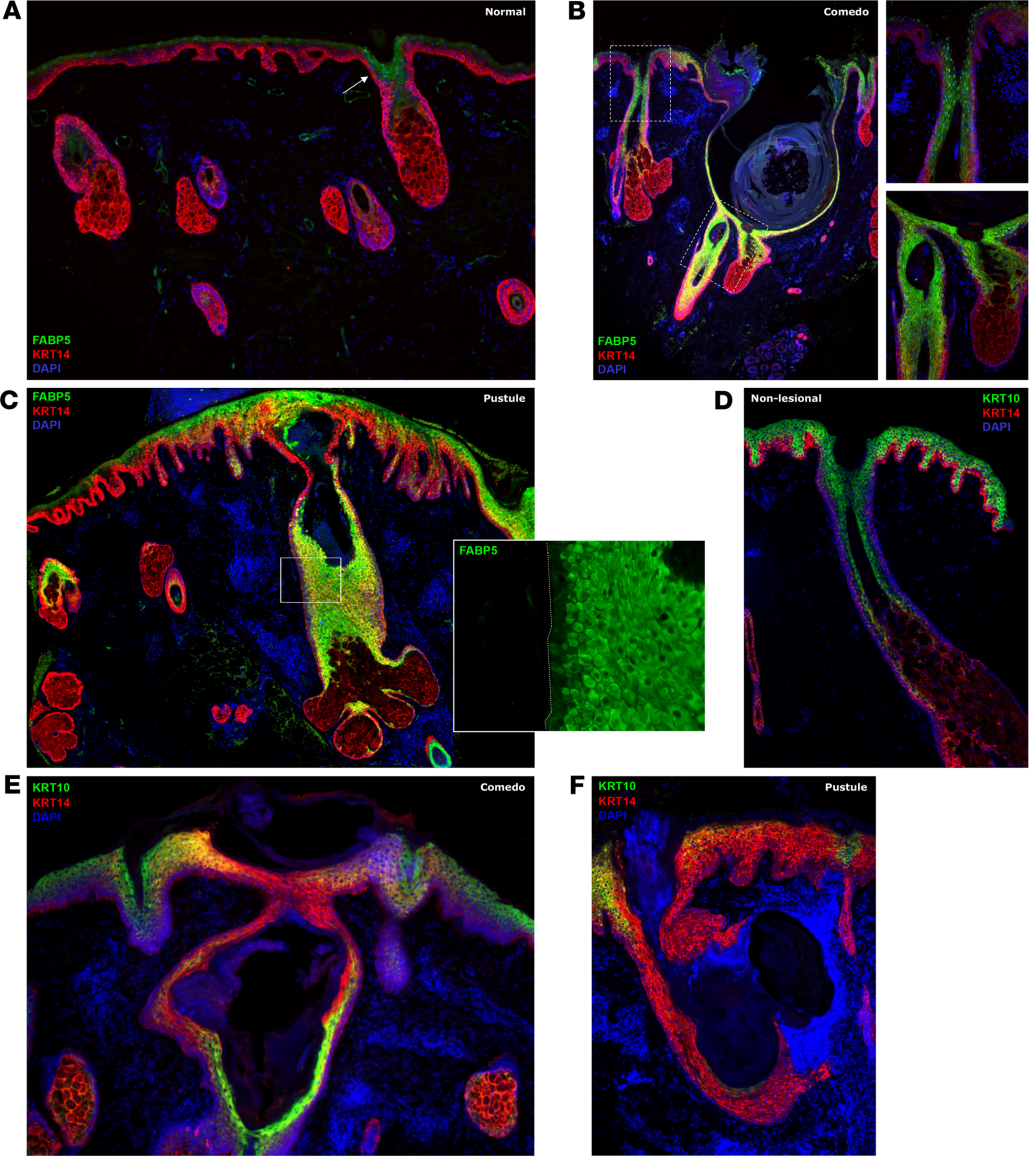
FABP5 and KRT10 in healthy and acne skin. (**A**) FABP5 (green) and KRT14 (red) in normal facial skin. The upper hair follicle expresses FABP5 (arrow). (**B**) FABP5 and KRT14 in comedonal specimen. Insets, magnified views of an adjacent hair follicle opening (top) and lower comedonal wall/hair follicle isthmus, with reduced imaging exposure (bottom). (**C**) Broad expression of FABP5 in a pustular lesion, including patchy basal layer expression (inset shows single channel view; the dotted line indicates the interface between the epidermis and dermis). (**D**) KRT10 (green) and KRT14 (red) in nonlesional facial skin from an acne patient. (**E**) Increased KRT10 in comedonal skin. (**F**) Reduced KRT10 in a pustular lesion. Original magnification, ×100; ×100–×200 (higher magnification images).

We next examined FABP5 protein levels in healthy and lesional skin by IHC. Consistent with our spatial transcriptomics data, FABP5 in healthy skin is expressed in the upper stratum spinosum, stratum granulosum, and differentiated cells of the hair follicle infundibulum (Figure 5A). In comedones, we observed increased FABP5 in comedonal walls (Figure 5B). In pustular acne lesions, FABP5 was broadly elevated in the abnormal follicular epithelium and the perilesional epidermis, suggesting generalized alterations in KC stress response and differentiation programs (Figure 5C). At both sites, expanded FABP5 expression included patchy involvement of the basal layer, consistent with our spatial profiling results (Figure 5C and Figure 4, F and G).

Also in agreement with our spatialomics data, we observed by IHC that KRT10 is increased in comedonal KCs (Figure 4D and Figure 5, D and E), consistent with the well-described hyperkeratinization seen in acne comedones (40, 41). In contrast, in 2 of 4 independent pustule specimens, KRT10 was reduced in both follicular epithelium and in the adjacent interfollicular epidermis (IFE), concordant with our transcriptomic findings pointing to an expansion of a dedifferentiated, basal-like cell state (Figure 4, D and E, and Figure 5F).

### AP-1 upregulates FABP5.

**Figure 6:**
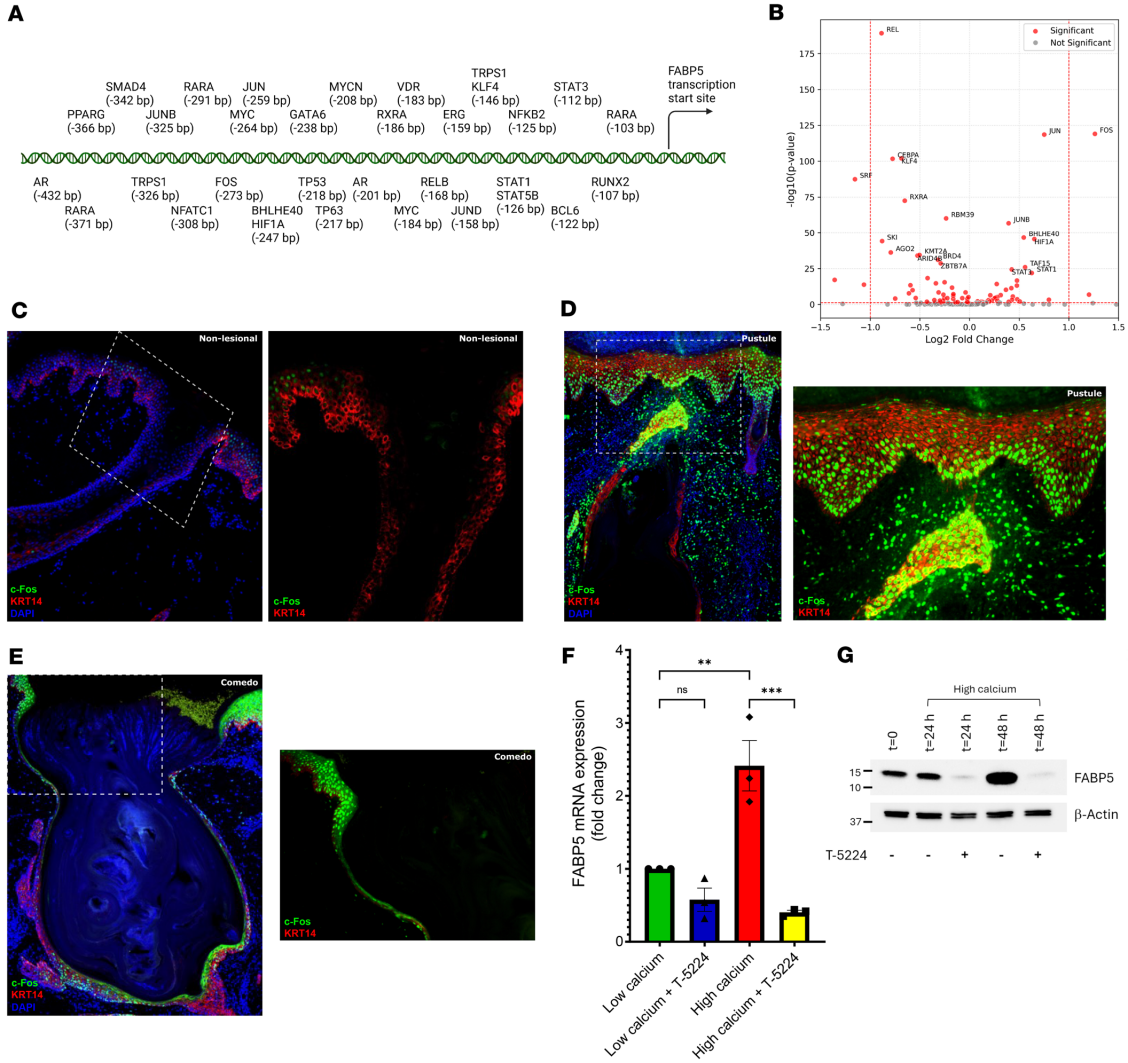
Regulation of FABP5 in human keratinocytes. (**A**) Schematic of transcription factors with binding motifs within 500 bp upstream of the *FABP5* transcriptional start site. (**B**) Expression of these transcription factors in keratinocytes from Do et al., comparing inflammatory acne lesions versus nonlesional skin, visualized by volcano plot (26). (**C**) Nonlesional skin stained for c-Fos (green) and KRT14 (red). The right panel is a magnified view without DAPI. (**D**) Pustular skin stained for c-Fos and KRT14. (**E**) Comedo stained for c-Fos and KRT14. The right panel is a magnified view without DAPI. (**F**) Relative *FABP5* mRNA levels in human N/TERT keratinocytes grown in low- or high-calcium media and treated with T-5224 (5 μM) or vehicle. (**G**) Western blot for FABP5 in N/TERT keratinocytes cultured in low-calcium media or shifted to high-calcium media, and treated with the AP-1 inhibitor T-5224 (5 μM) or vehicle, for the indicated hours. Statistical significance was determined using a 1-way ANOVA with post hoc *t* test and Tukey’s correction for multiple comparisons (**P < 0.01; ***P < 0.001). Data are shown as mean ± SEM. Original magnification, ×100; ×100–x200 (higher magnification images).

To understand how *FABP5* expression is modulated, we searched the Gene Transcription Regulation Database (GTRD) for transcription factor (TF) binding sites within 500 bp upstream of the *FABP5* transcriptional start site (TSS) (42). Among TFs with recognitions sites in this region (Supplemental Table 3), we found several that are expressed in the skin and may be functionally important for cutaneous inflammation, differentiation, and homeostasis (Figure 6A). These include TFs previously implicated in hair follicle homeostasis (GATA6, RUNX2, TRPS1, MYCN) (14, 43–45), barrier function (KLF4) (46), basal layer homeostasis (TP63, MYC) (47, 48), lipid responses (PPARG) (49), KC differentiation (FOS, JUN, JUNB, RARA) (50, 51), and cytokine, hormone, and growth factor responses (AR, NFATC1, STAT1, STAT3, STAT5B, SMAD4, VDR) (52).

Using published scRNA-Seq data from inflammatory acne lesions (26), we next systematically queried the expression of all TFs (*n* = 160) identified above. We observed that the top 3 upregulated TF genes ranked by –log_10_(*P* value) were *FOS*, *JUN*, and *JUNB*, which all encode members of the activator protein 1 (AP-1) complex (Figure 6B). Consistent with these data, IHC showed increased nuclear c-Fos in follicular KCs and adjacent IFE in both acne comedones and pustules compared with healthy skin (Figure 6, C–E).

Previous studies have shown that both AP-1 and FABP5 are induced following calcium switch-mediated KC differentiation (53, 54). Using human N/TERT KCs, we confirmed that FABP5 mRNA and protein levels increased after 48 hours of culture in high calcium media (Figure 6, F and G). Addition of T-5224, a small molecule inhibitor of c-Fos/AP-1 (55), severely inhibited the upregulation of FABP5 mRNA and protein in response to high calcium (Figure 6, F and G). Altogether, these findings suggest that increased AP-1 in acne lesions potentiates the upregulation of FABP5, which in turn reduces RAR signaling, ultimately leading to a progrowth/antidifferentiation phenotype.

### Topical AP-1 inhibition decreases pustule formation in a mouse model of folliculitis.

Neutrophil infiltration is commonly observed in inflammatory acne. Since we noted an influx of neutrophils and other myeloid cells in pustules (Figure 2F and Supplemental Figure 5, A–D), we therefore examined whether disease-associated KC and sebocyte populations express known potentiators of myeloid infiltration or function. Indeed, we observed that *CXCL8*, a potent chemokine that attracts neutrophils, is elevated in multiple KC populations in comedonal lesions (Supplemental Figure 5, E and F). In pustular KCs, both *CXCL8* and *MMP1* (encoding a matrix metalloproteinase also implicated in myeloid cell recruitment) are elevated (Supplemental Figure 5, F and G) (56, 57).

**Figure 7:**
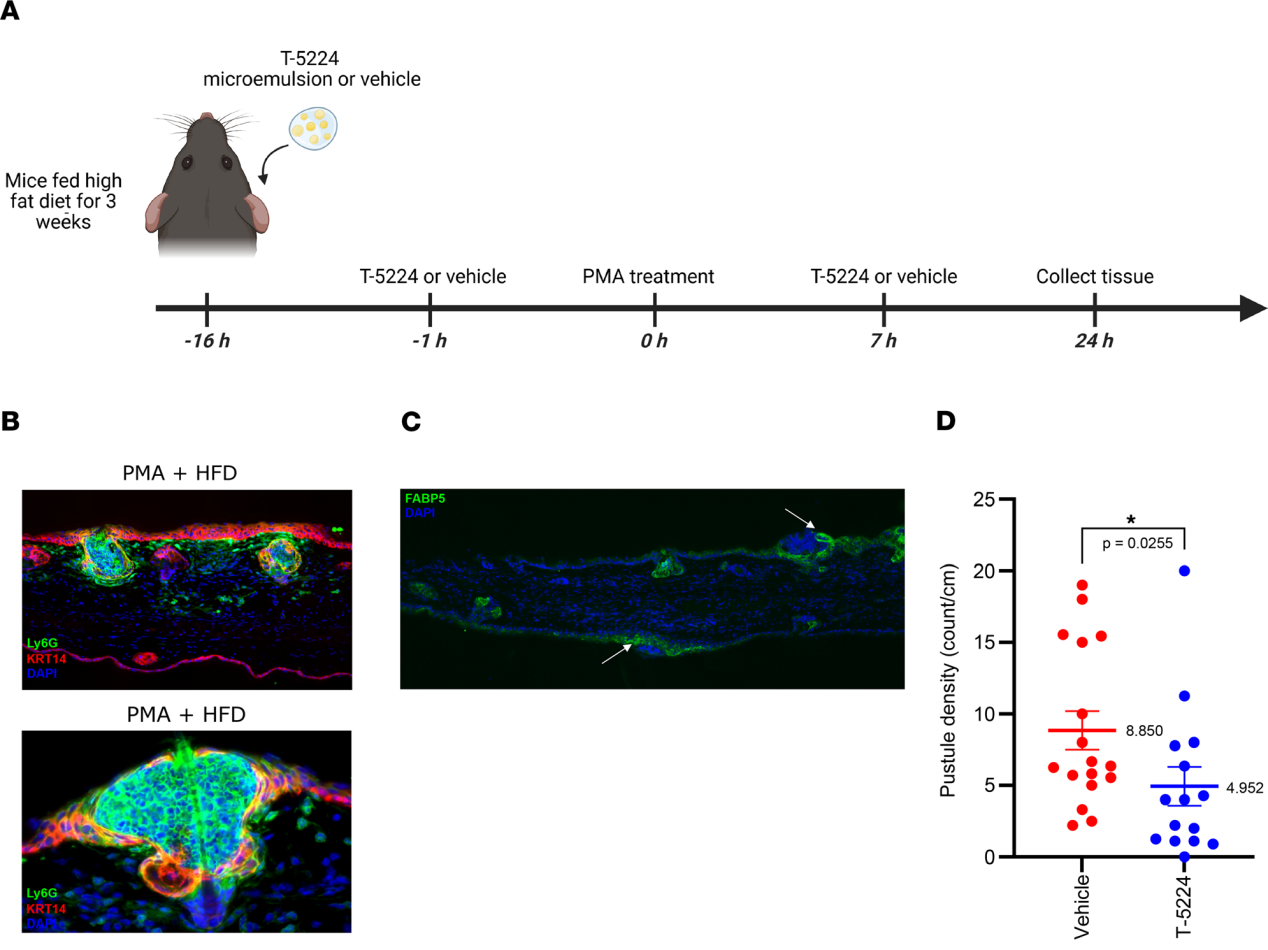
AP-1 inhibition reduces abscess formation in a mouse model of neutrophilic folliculitis. (**A**) Schematic of experimental design. Mice were fed a high-fat diet (HFD) for 3 weeks and then treated with topical T-5224 (0.25%) or vehicle, both before and after PMA application to the ear. Ear tissue was harvested 24 hours after PMA for analysis. (**B**) Representative low- and higher-magnification images of ear skin from HFD-fed mice treated with PMA, stained for the neutrophil marker Ly6G (green) and KRT14 (red), showing prominent neutrophilic pustules. (**C**) FABP5 (green) is increased in keratinocytes near neutrophilic pustules (arrows). (**D**) Quantification of pustule density showing a significant reduction in T-5224-treated mice (*P* = 0.0255). Statistical significance was determined using a Mann-Whitney test (*P < 0.05). Data are shown as mean ± SEM. Original magnification, ×200 (**B**, top), ×400 (**B**, bottom), ×100 (**C**).

A previous study has shown that mice fed a high-fat diet (HFD), followed by topical application of phorbol 12-myristate 13-acetate (PMA), acquire a neutrophilic folliculitis whose gene expression signature resembles acne (58). Neutrophil recruitment in this model is dependent on hair follicle KC-derived *CXCL1*, a murine ortholog to human *CXCL8* (58). We confirmed that HFD-fed mice develop neutrophilic folliculitis following PMA exposure, and further observed that epidermal KCs surrounding these neutrophilic pustules display increased FABP5 compared with uninvolved skin (Figure 7, A–C).

Given our earlier findings showing that AP-1 is increased in acne and can modulate FABP5 expression in vitro, we next tested whether AP-1 inhibition can suppress pustule formation in this model. For these experiments, we topically applied T-5224 microemulsion (*n* = 15) or vehicle (*n* = 17) onto the ears of HFD-fed mice prior to and after PMA challenge (Figure 7A). In vehicle-treated mice, PMA elicited marked neutrophil accumulation in follicles and pustule formation (Figure 7D). In contrast, T-5224 treatment reduced pustule formation by 44.0% compared with vehicle-treated controls (mean pustule density = 8.85/cm for vehicle versus 4.95/cm for T-5224, *P* = 0.0255) (Figure 7D). These data suggest that topical AP-1 inhibition can suppress neutrophilic infiltration and pustule formation in mice and may therefore provide a strategy for treating inflammatory acne in patients.

## Discussion

Sebaceous gland-KC interactions are thought to modulate acne pathogenesis. To date, the difficulty of capturing sebocytes from fresh tissue for scRNA-Seq has hindered investigations into the functional role of the sebaceous gland compartment in acne. Here, we confirm that basal sebocytes are transcriptionally heterogenous, with both PPARG^+^ and PPARG^–^ subpopulations. PPARG^–^ cells are more proliferative, supporting the model that basal sebaceous cells progressively differentiate through a KRT5^+^PPARG^+^ transitional cell state. In comedo-associated sebaceous glands, we find increased expression of key enzymes in sebum production. In contrast, pustule-associated sebaceous glands exhibit lower expression of lipogenic enzymes, raising the possibility that an inflammatory milieu may suppress sebocyte homeostasis and lipid synthesis.

Our creation of a custom *KRT5*-driven segmentation routine was critical for accurately assigning transcripts to basal sebocytes. While this segmentation approach was the most restrictive of the 4 methods we compared, it also yielded cell contours that most closely mirrored the spindled shapes of peripheral sebocytes seen by histology and had the best performance at excluding improperly assigned transcripts. This simple approach can readily be adapted to incorporate other marker genes to help define cellular boundaries for complex tissues in which strict exclusion of inappropriate transcripts is required.

A limitation of this study is our use of a targeted 100-gene panel. This strategy is subject to unavoidable bias in gene selection, which limits the discovery of pathways not previously associated with KC or sebocyte function. For example, our panel lacked specific markers to definitively subtype myeloid populations, preventing us from clearly distinguishing the dense neutrophilic infiltrate seen by H&E staining from other myeloid cells in our spatial analysis. While the Xenium platform now offers panels of > 5,000 genes, these were not available when we began our study. Notably, smaller panels have the advantage of providing greater sensitivity in detecting less-abundant transcripts, as these panels utilize up to 8 probes per gene target while larger panels typically use 2–3 probes per target (59). Therefore, for applications where high sensitivity is required, a targeted panel may be preferable. Another limitation is the age mismatch between our healthy controls (ages 27–49) and the patients with acne (18–33). While our use of paired nonlesional skin (where available) from patients with acne helped control for individual-specific factors, future studies incorporating age-matched healthy controls would be valuable for confirming these findings.

Our data lend support to the model that comedogenesis is accompanied by loss of follicular identity and a shift toward an IFE-like phenotype. In comedones, we observed loss of *GATA6*, which in mice causes aberrant differentiation in the upper hair follicle (14, 60, 61). Loss of *KRT79* in comedonal KCs is also consistent with our previously published data (15).

Currently, oral retinoids such as isotretinoin are the most effective therapies for acne. Isotretinoin improves acne in > 90% of patients, and approximately two-thirds of responders will have durable remissions (62). However, retinoids can potentiate dramatic flares (acne fulminans, including systemic symptoms such as fever and arthritis) in the most inflammatory forms of acne (63). Moreover, inflammatory pustular lesions are thought to be less responsive to topical retinoid monotherapy than are comedonal lesions (64).

In our data, sebocytes and follicular KCs from both comedonal and pustular samples display gene expression changes consistent with decreased retinoid signaling, in agreement with previous reports (13). We also observed an increased *FABP5*/*CRABP2* ratio in both comedones and pustules, which is predicted to increase PPAR-β/δ signaling at the expense of RAR signaling in response to retinoic acid, leading to hyperproliferation (13). In pustules, the *FABP5*/*CRABP2* ratio was especially high in basal layer cells. Given these alterations, intracellular retinoic acid transporters such as FABP5 and CRABP2 deserve further investigation as potential mediators of paradoxical responses to retinoids in inflammatory acne. We also observed decreased retinoid receptor (*RARG* and *RXRA*) transcript expression in both comedones and pustules compared with nonlesional skin. This presents an apparent paradox, as retinoids are highly effective therapies for acne. However, retinoids primarily prevent new acne lesion formation (and can acutely exacerbate existing inflammatory lesions), so nonlesional skin may be their most important site of therapeutic action (65). Moreover, if untreated acne lesions at baseline possess a relative deficiency of retinoid receptor expression, as our data and others suggest (26), then pharmacologic retinoids may act to restore a more normal signaling and transcriptional profile.

Apart from its role in retinoid signaling, FABP5 can also promote KC differentiation and inflammatory responses. As an inflammatory mediator, FABP5 interacts with valosin-containing protein (VCP) in KCs to promote NF-κB activation and the production of neutrophil chemoattractants (66). Our data suggest that treatment with T-5224, a potent AP-1 inhibitor, may be a strategy for decreasing *FABP5* expression, and our analysis of TF binding sites suggests additional candidates for investigation.

Since *FABP5* is upregulated in other skin diseases such as psoriasis, it is possible that this gene is part of a general KC stress response program. Under inflammatory conditions, KCs often display reduced KRT10, increased KRT5/14, and increased expression of stress-associated keratins (K6, K16), consistent with gene expression patterns we observed in pustules (Figure 4E) (67). Importantly, we also observed elevated FABP5 in the hyperkeratinized, KRT10^hi^ comedonal KCs, suggesting that noninflammatory lesions may also upregulate FABP5 independently of the KC stress response. Since sebum is rich in fatty acids that are excreted into the upper hair follicle, where FABP5 expression is especially prevalent, a role for this protein in modulating follicular homeostasis and inflammatory follicular occlusion disorders is an intriguing possibility.

Our findings in a mouse model of folliculitis suggest that AP-1 activity is required for the formation of pustular lesions. We propose that inhibiting AP-1 may reduce the expression of KC inflammatory modules, including genes encoding neutrophil chemoattractants. Our in vivo findings support the translational potential of targeting AP-1 as an alternative or adjunctive approach to retinoid therapy. Unlike systemic retinoids, which carry risks of paradoxical flares, dryness, liver injury, and hyperlipidemia (1), topical AP-1 inhibitors such as T-5224 may offer a strategy to suppress inflammation and normalize retinoid signaling.

Altogether, our findings are consistent with a model in which lipid metabolism, sebocyte and KC differentiation, and retinoid responses vary along the noninflammatory to inflammatory acne lesional spectrum. The relative deficiency of retinoid signaling in acne lesional skin provides a strong rationale for restoring this pathway by pharmacologically rebalancing FABP5/CRABP2 ratios. Drugs that inhibit FABP5 or AP-1 may therefore represent candidate therapies for treating this common skin disorder.

## Methods

### Sex as a biological variable.

Patient demographics, including sex and age, are detailed in Supplemental Table 1. Due to the limited sample size, sex-based comparisons of human transcriptomic data were not performed. In vivo mouse studies utilized male and female mice in similar numbers for both treatment groups. All in vitro experiments were performed using the N/TERT human KC cell line.

### Human skin samples.

Acne donors and healthy controls were recruited by the University of Michigan’s Program for Clinical Research in Dermatology (PCRiD). Individuals were excluded if they were undergoing acne treatment or hormone regulating therapies. We collected 4 mm punch biopsies of normal skin, comedones, or pustules. Comedones included both open and closed lesions, which were analyzed together as a single group. Pustules were follicularly centered papules with erythema or purulence, while comedones were noninflamed smooth papules with follicular plugging. Acne severity scores were not uniformly collected for all archival specimens. Specimens were fixed in 10% neutral buffered formalin overnight and processed as formalin-fixed paraffin embedded (FFPE) blocks. For Xenium in situ analysis, 5 μm sections were prepared under RNase-free conditions, mounted onto Xenium analyzer slides (10X Genomics), and submitted to the Advanced Genomics Core (AGC) at the University of Michigan for processing. The Xenium multimodal cell segmentation add-on kit was used for all but 4 sections (all archival), which were processed prior to the release of the kit. Visualization of Xenium cell segmentation boundaries, transcripts, and alignment of transcript coordinates with scanned H&E-stained sections were performed using Xenium Explorer 3 software (10X Genomics).

### Cell segmentation, clustering, and cell type annotation.

The Python package Scanpy (v1.10) was used for Xenium and scRNA-Seq data processing, including quality control, transcript count normalization, log transformation, and differential gene expression analysis (68). For Xenium analysis, cells with fewer than 10 transcripts were excluded. All samples were merged into a single expression matrix, and unsupervised clustering (Leiden algorithm) was used to identify cell type populations. Squidpy (v1.6.2) was used to create spatial plots of cell coordinates. Resegmentation of spatial transcriptomic datasets was performed using the Xenium Ranger software for the nuclei expansion (2 μm and 5 μm) approaches. For all segmentation approaches, visualization of cell boundaries, transcripts, and alignment with H&E-stained sections were performed using Xenium Explorer 3 software (10X Genomics).

For *KRT5*-directed resegmentation, the default cell boundaries from the Xenium analyzer were imported into Python using the spatialdata_io (v0.1.6) package (69). Both cell boundaries and transcript coordinate matrices were converted to geopandas.GeoDataFrame (v1.0.1) objects to facilitate geometrical operations. Any holes detected in cell boundary or nucleus polygons were filled using the concave_hull command from the shapely library (v2.0.6). Cell polygons containing at least 3 *KRT5* transcripts were flagged for resegmentation. For each resegmented cell, the shapely.convex_hull command was used to draw a minimal polygon containing all *KRT5* transcripts. The *KRT5* polygon was merged with the cell nucleus polygon using shapely.unary_union, creating the new cell boundary. Transcripts were reassigned based on the new cell boundary polygons, and new Xenium output files were created using the sopa API (v.1.1.6) (70).

### Immunofluorescence staining.

FFPE sections were rehydrated and boiled in antigen retrieval buffer (1 mM EDTA in water, pH 8.0), blocked with 20% normal donkey serum, and probed with antibodies (Supplemental Table 4) against the following antigens: c-FOS (1:100, Cell Signaling), FABP5 (1:500, Cell Signaling), Ki67 (1:100, Cell Signaling and BD Biosciences), KRT5 (1:2,000, BioLegend), KRT10 (1:2,000, Covance), KRT14 (1:2,000, BioLegend), KRT79 (1:2,000, Santa Cruz), and PPARG (1:300, Cell Signaling, amplified using the TSA Fluorescein Plus kit). The fluorescent images were processed with Adobe Photoshop CS6, using the autoblend feature to automatically maximize image sharpness across multiple focal planes.

### RNA isolation and qPCR analysis.

For qPCR, 2.5 × 10^5^ N/TERT cells per well were plated in a 12-well plate in KC-serum free medium (K-SFM, Gibco) supplemented with bovine pituitary extract (BPE, 25 μg/mL, Invitrogen), L-glutamine (2 mM, Invitrogen), EGF (0.2 ng/mL, Invitrogen), and CaCl_2_ (300 μM, Sigma-Aldrich) (71). After overnight culture, the cells were changed to fresh K-SFM or to high calcium media (1.8 mM) as previously described (71). Media were supplemented with T-5224 (5 μM, Tocris Bioscience) or DMSO vehicle (0.1% final concentration). After 48 hours, RNA was extracted using the RNeasy kit (Qiagen) and reverse transcribed using the High-Capacity cDNA Reverse Transcription kit (Applied Biosystems). qPCR was performed using Power SYBR Green PCR Master Mix (Applied Biosystems). Primers are as follows: *GAPDH* forward (5′-CGTAGACAAAATGGTGAAGGTCGG-3′); *GAPDH* reverse (5′-AAGCAGTTGGTGGTGCAGGATG-3′); *FABP5* forward (5′-TGAAGGAGCTAGGAGTGGGAA-3′); and *FABP5* reverse (5′-TGCACCATCTGTAAAGTTGCAG-3′).

### Western blotting.

For each condition, 7 × 10^5^ N/TERT cells per well were plated in a 6-well plate. When confluent, cells were switched to high calcium media containing T-5224 (5 μM, Tocris Bioscience) or DMSO vehicle (0.1% final concentration) for 24 or 48 hours before collecting lysates. Protein lysates were prepared with Laemmli buffer (Bio-Rad Laboratories) containing protease inhibitor (Sigma-Aldrich) and PhosSTOP (Roche), and they were probed with antibodies against FABP5 (1:1,000, Cell Signaling Technology) and β-actin (1:10,000, Cell Signaling Technology, #12262). Images were captured using a ChemiDoc system (Bio-Rad Laboratories).

### Mouse model of neutrophilic folliculitis.

To prepare the T-5224 microemulsion, we first created a 1:2:2 mixture of oleic acid (ThermoFisher, 31997.14), CARBITOL (diethylene glycol monoethyl ether, Sigma, W509043-1KG), and polysorbate 20 (Fisher, BP337-100), respectively (72). After vortexing, 3 mL of this mixture was used to dissolve 10 mg of T-5224 (Tocris Bioscience). In total, 1 mL of deionized water was then added, dropwise with vortexing, to this solution to obtain a final volume of 4 mL and a T-5224 concentration of 0.25% (w/v). Fresh T-5224 microemulsions were prepared for each experiment and stored at room temperature between applications. The vehicle control was prepared and stored similarly.

Starting at 5–6 weeks of age, mice of a C57BL/6J background (Jackson Laboratory) were fed high-fat chow (60% kcal fat, Research Diets, D12492) for 3 weeks (58). The dorsal ear was pretreated with the T-5224 microemulsion (50 μL) or vehicle, followed 16 hours later by 20 μL of 10 μg/mL PMA (phorbol 12-myristate 13-acetate, Selleckchem, S7791) in acetone. Treatment with T-5224 or vehicle was repeated at 1 hour before and 7 hours after PMA application. Tissue was harvested 24 hours after PMA application, fixed in 3.7% paraformaldehyde at 4°C for 1 hour, rinsed in PBS, incubated in 30% sucrose/PBS overnight at 4°C, and embedded into OCT (Avantor, 25608-930). Frozen sections were rehydrated in PBS and probed with antibodies to Ly-6G (127601, 1:500, BioLegend), FABP5, or KRT14. To quantitate pustule density, we counted perifollicular and intraepidermal clusters containing at least 25 Ly6G^+^ cells across tissue sections and divided the number of clusters by the length of the section. All experiments were performed in accordance with guidelines established by the University of Michigan Unit for Laboratory Animal Medicine (study protocol no. PRO00011782).

### Statistics.

Statistical analyses were performed using GraphPad Prism version 10, and *P* ≤ 0.05 was considered significant. For comparisons involving more than 2 groups, we used a 1-way ANOVA with post hoc 2-tailed *t* test, and Tukey’s correction was used for multiple comparisons, unless otherwise stated in the figure legend. Data are shown as 95% CI unless otherwise stated.

### Study approval.

This study was performed in accordance with protocols (HUM00070357, HUM003422, and HUM00174864) approved by the IRB at the University of Michigan. All participants provided written, informed consent.

### Data availability.

Spatial transcriptomics data are available on GEO (GSE301280). We have made code publicly available in the joe-durgin/TranscriptFocusedSegmentation Github repository (https://github.com/joe-durgin/TranscriptFocusedSegmentation; commit ID 35b6c02). Supporting data values for all graphs and charts presented in the figures are available in the Supporting Data Values file.

## Author contributions

SYW and JSD conceived and designed the study. JEG provided input on study and experimental design. JEG and JF provided human samples. JSD developed the *KRT5*-based segmentation pipeline and performed all analyses. YC and LCT provided alternative segmentation methods for comparison. JSD, TJH, and NAV performed IHC. JSD, SYT, MKS, and TJH performed in vitro studies. NAV, JSD, and TJH performed the mouse studies. SYW supervised the project. JSD and SYW wrote the manuscript. All authors reviewed and approved the final version of the manuscript.

## Funding support

This work is the result of NIH funding, in whole or in part, and is subject to the NIH Public Access Policy. Through acceptance of this federal funding, the NIH has been given a right to make the work publicly available in PubMed Central.

- NIH (T32AR007197; to JSD)
- NIH (R01AR080654 and R01AR065409; to SYW)
- P30AR075043
- P30CA046592 through the Single Cell and Spatial Analysis Shared Resource
- at the University of Michigan

## Supplementary Material

Supplemental data

Unedited blot and gel images

Supporting data values

## Version 1 02/09/2026

Electronic publication

## References

[1] Reynolds RV (2024). Guidelines of care for the management of acne vulgaris. J Am Acad Dermatol.

[2] Hay RJ (2014). The global burden of skin disease in 2010: an analysis of the prevalence and impact of skin conditions. J Invest Dermatol.

[3] Lim HW (2017). The burden of skin disease in the United States. J Am Acad Dermatol.

[4] Mallon E (1999). The quality of life in acne: a comparison with general medical conditions using generic questionnaires. Br J Dermatol.

[5] Owen CE (2014). Treating acne with high-dose isotretinoin. JAMA.

[6] Dreno B (2024). Acne microbiome: From phyla to phylotypes. J Eur Acad Dermatol Venereol.

[7] Deng Y (2024). Skin barrier dysfunction in acne vulgaris: pathogenesis and therapeutic approaches. Med Sci Monit.

[8] Cong T (2019). From pathogenesis of acne vulgaris to anti-acne agents. Arch Dermatol Res.

[9] Zouboulis CC (2020). Endocrinology and immunology of acne: Two sides of the same coin. Exp Dermatol.

[10] Firlej E (2022). The role of skin immune system in acne. J Clin Med.

[11] Thiboutot DM (2008). Overview of acne and its treatment. Cutis.

[12] Schneider AM (2023). Evolution of the facial skin microbiome during puberty in normal and acne skin. J Eur Acad Dermatol Venereol.

[13] Melnik BC (2023). Acne transcriptomics: fundamentals of acne pathogenesis and isotretinoin treatment. Cells.

[14] Oulès B (2020). Contribution of GATA6 to homeostasis of the human upper pilosebaceous unit and acne pathogenesis. Nat Commun.

[15] Veniaminova NA (2013). Keratin 79 identifies a novel population of migratory epithelial cells that initiates hair canal morphogenesis and regeneration. Development.

[16] Stathakis V (1997). Descriptive epidemiology of acne vulgaris in the community. Australas J Dermatol.

[17] Ludovici M (2018). Influence of the sebaceous gland density on the stratum corneum lipidome. Sci Rep.

[18] Zouboulis CC (2022). Sebaceous immunobiology-skin homeostasis, pathophysiology, coordination of innate immunity and inflammatory response and disease associations. Front Immunol.

[19] Lovászi M (2017). Sebaceous-immunobiology is orchestrated by sebum lipids. Dermatoendocrinol.

[20] Shi VY (2015). Role of sebaceous glands in inflammatory dermatoses. J Am Acad Dermatol.

[21] Zouboulis CC (2006). Isotretinoin revisited: pluripotent effects on human sebaceous gland cells. J Invest Dermatol.

[22] Nelson AM (2006). 13-cis Retinoic acid induces apoptosis and cell cycle arrest in human SEB-1 sebocytes. J Invest Dermatol.

[23] Schug TT (2007). Opposing effects of retinoic acid on cell growth result from alternate activation of two different nuclear receptors. Cell.

[24] Seukeran DC, Cunliffe WJ (1999). The treatment of acne fulminans: a review of 25 cases. Br J Dermatol.

[25] Trivedi NR (2006). Gene array expression profiling in acne lesions reveals marked upregulation of genes involved in inflammation and matrix remodeling. J Invest Dermatol.

[26] Do TH (2022). TREM2 macrophages induced by human lipids drive inflammation in acne lesions. Sci Immunol.

[27] Kang S (2005). Inflammation and extracellular matrix degradation mediated by activated transcription factors nuclear factor-kappaB and activator protein-1 in inflammatory acne lesions in vivo. Am J Pathol.

[28] Deng M (2024). Analysis of intracellular communication reveals consistent gene changes associated with early-stage acne skin. Cell Commun Signal.

[29] O’Neill AM (2022). Antimicrobial production by perifollicular dermal preadipocytes is essential to the pathophysiology of acne. Sci Transl Med.

[30] Thalheim T, Schneider MR (2024). Skin single-cell transcriptomics reveals a core of sebaceous gland-relevant genes shared by mice and humans. BMC Genomics.

[31] Schmidt M (2024). A spatial portrait of the human sebaceous gland transcriptional program. J Biol Chem.

[32] Seiringer P (2024). Spatial transcriptomics reveals altered lipid metabolism and inflammation-related gene expression of sebaceous glands in psoriasis and atopic dermatitis. Front Immunol.

[34] Veniaminova NA (2023). Distinct mechanisms for sebaceous gland self-renewal and regeneration provide durability in response to injury. Cell Rep.

[35] Veniaminova NA (2019). Niche-specific factors dynamically regulate sebaceous gland stem cells in the skin. Dev Cell.

[37] Salas SM (2025). Optimizing Xenium In Situ data utility by quality assessment and best-practice analysis workflows. Nat Methods.

[38] Fischer H (2017). Holocrine secretion of sebum is a unique DNase2-dependent mode of programmed cell death. J Invest Dermatol.

[39] Levi L (2015). Saturated fatty acids regulate retinoic acid signalling and suppress tumorigenesis by targeting fatty acid-binding protein 5. Nat Commun.

[40] Plewig G (1974). Follicular keratinization. J Invest Dermatol.

[41] Thiboutot DM (2000). The role of follicular hyperkeratinization in acne. J Dermatol Treat.

[42] Kolmykov S (2021). GTRD: an integrated view of transcription regulation. Nucleic Acids Res.

[43] Glotzer DJ (2008). Impaired skin and hair follicle development in Runx2 deficient mice. Dev Biol.

[44] Fantauzzo KA (2008). Dynamic expression of the zinc-finger transcription factor Trps1 during hair follicle morphogenesis and cycling. Gene Expr Patterns.

[45] Trieu KG (2022). Basal cell carcinomas acquire secondary mutations to overcome dormancy and progress from microscopic to macroscopic disease. Cell Rep.

[46] Segre JA (1999). Klf4 is a transcription factor required for establishing the barrier function of the skin. Nat Genet.

[47] Truong AB, Khavari PA (2007). Control of keratinocyte proliferation and differentiation by p63. Cell Cycle.

[48] Gebhardt A (2006). Myc regulates keratinocyte adhesion and differentiation via complex formation with Miz1. J Cell Biol.

[49] Chinetti G (2000). Peroxisome proliferator-activated receptors (PPARs): nuclear receptors at the crossroads between lipid metabolism and inflammation. Inflamm Res.

[50] Briata P (1993). AP-1 activity during normal human keratinocyte differentiation: evidence for a cytosolic modulator of AP-1/DNA binding. Exp Cell Res.

[51] Vollberg Sr TM (1992). Retinoic acid receptors as regulators of human epidermal keratinocyte differentiation. Mol Endocrinol.

[52] Takeda K, Akira S (2000). STAT family of transcription factors in cytokine-mediated biological responses. Cytokine Growth Factor Rev.

[53] Saeki Y (2012). An ErbB receptor-mediated AP-1 regulatory network is modulated by STAT3 and c-MYC during calcium-dependent keratinocyte differentiation. Exp Dermatol.

[54] Bikle DD (2003). Vitamin D regulated keratinocyte differentiation: role of coactivators. J Cell Biochem.

[55] Uchihashi S (2011). Metabolism of the c-Fos/activator protein-1 inhibitor T-5224 by multiple human UDP-glucuronosyltransferase isoforms. Drug Metab Dispos.

[56] Shao S (2016). Increased lipocalin-2 contributes to the pathogenesis of psoriasis by modulating neutrophil chemotaxis and cytokine secretion. J Invest Dermatol.

[57] Estrada-Gutierrez G (2011). Increased expression of matrix metalloproteinase-1 in systemic vessels of preeclamptic women: a critical mediator of vascular dysfunction. Am J Pathol.

[58] Nakamizo S (2021). High-fat diet induces a predisposition to follicular hyperkeratosis and neutrophilic folliculitis in mice. J Allergy Clin Immunol.

[59] https://cdn.10xgenomics.com/image/upload/v1721078232/support-documents/CG000775_Prime5K_DataHighlightsTN_RevA.pdf#.

[60] Swanson JB (2019). Loss of Gata6 causes dilation of the hair follicle canal and sebaceous duct. Exp Dermatol.

[61] Donati G (2017). Wounding induces dedifferentiation of epidermal Gata6^+^ cells and acquisition of stem cell properties. Nat Cell Biol.

[62] Blasiak RC (2013). High-dose isotretinoin treatment and the rate of retrial, relapse, and adverse effects in patients with acne vulgaris. JAMA Dermatol.

[63] Li AW, Antaya RJ (2018). Isotretinoin-induced acne fulminans without systemic symptoms with concurrent exuberant granulation tissue. Pediatr Dermatol.

[64] Zaenglein AL (2016). Guidelines of care for the management of acne vulgaris. J Am Acad Dermatol.

[65] Leung AK (2021). Dermatology: how to manage acne vulgaris. Drugs Context.

[66] Hao J (2023). Keratinocyte FABP5-VCP complex mediates recruitment of neutrophils in psoriasis. Cell Rep.

[67] Zhang X (2019). Keratin 6, 16 and 17—critical barrier alarmin molecules in skin wounds and psoriasis. Cells.

[68] Wolf FA (2018). SCANPY: large-scale sin gle-cell gene expression data analysis. Genome Biol.

[69] Marconato L (2024). SpatialData: an open and universal data framework for spatial omics. Nat Methods.

[70] Blampey Q (2024). Sopa: a technology-invariant pipeline for analyses of image-based spatial omics. Nat Commun.

[71] Smits JPH (2017). Immortalized N/TERT keratinocytes as an alternative cell source in 3D human epidermal models. Sci Rep.

[72] Bhatia G (2013). Adapalene microemulsion for transfollicular drug delivery. J Pharm Sci.

