# Supplementary material for: Spatial transcriptomics identifies differentiation, lipid metabolism, and retinoid pathway alterations in acne vulgaris
Source: JCI Insight. 2026 Feb 9;11(3):e198021. doi: 10.1172/jci.insight.198021 (PMC12892907; doi:10.1172/jci.insight.198021)
Supplement: Supplemental data [file jciinsight-11-198021-s115.pdf]

Figure S1. Immunohistochemistry of normal sebaceous glands.

A) Sebaceous glands from healthy skin stained for PPARG (green) and KRT5 (red) or Ki67 (red).

Figure S2. Comparison of segmentation methods in basal sebocytes.

A) Normalized *AWAT2* mRNA expression in basal sebocytes segmented using four different methods. Our custom *KRT5*-directed segmentation approach minimized *AWAT2* expression in basal sebocytes, suggesting improved accuracy of transcript assignment. Statistical significance was determined using one-way ANOVA with post-hoc t-tests and Tukey's correction for multiple comparisons (***$p < 0.001$, ****$p < 0.0001$).

Figure S3. Comparison of gene expression in paired patient samples. A) Normalized mRNA levels of key lipogenic (e.g., *FASN*) and retinoid pathway (e.g., *RARG*, *RARRES1*) genes in sebocytes from paired samples. **B)** Normalized mRNA levels of select genes in suprabasal keratinocytes from available paired non-lesional, comedonal, and/or pustular samples by patient.

Figure S4. Comparison of key hair follicle differentiation markers and retinoic acid response across acne disease states.

A–C) Normalized mRNA levels of *KRT79*, *GATA6*, and *RARRES1* in follicular keratinocytes across samples.

Figure S5. Recruitment of myeloid cells in acne skin.

A) Representative pustule from the right cheek of Patient 10 (H&E-stained section, scale bar = 2000 μm). **B)** Higher power view of the inflammatory infiltrate in the same lesion (scale bar = 200 μm). **C-D)** Proportion of myeloid and lymphocyte cells by disease state. Statistical significance was determined using a one-way ANOVA with post-hoc t-test and Tukey's correction for multiple comparisons (****, $p < 0.0001$). Error bars represent mean ± standard error. **E)** Normalized *CXCL8* expression in UMAPs across disease states. **F-G)** Normalized mRNA levels of *CXCL8* and *MMP1* in follicular keratinocytes across healthy, non-lesional, comedonal, and pustular conditions.

A

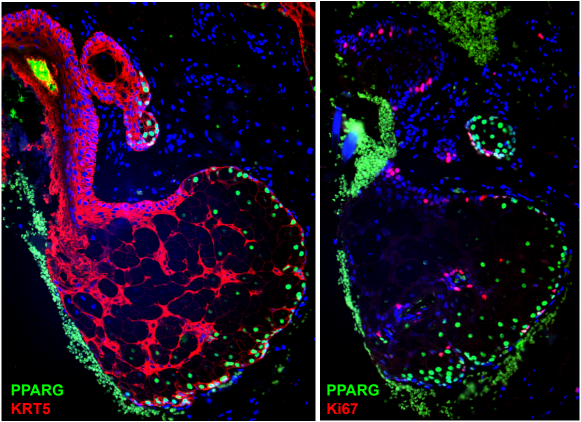

A

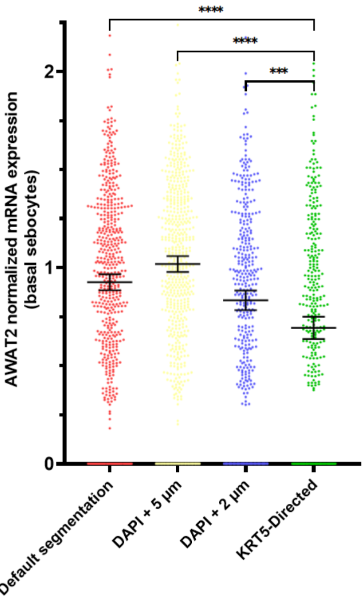

****
****
***
2
1
0
AWAT2 normalized mRNA expression
(basal sebocytes)
Default segmentation
DAPI + 5 µm
DAPI + 2 µm
KRT5-Directed

A

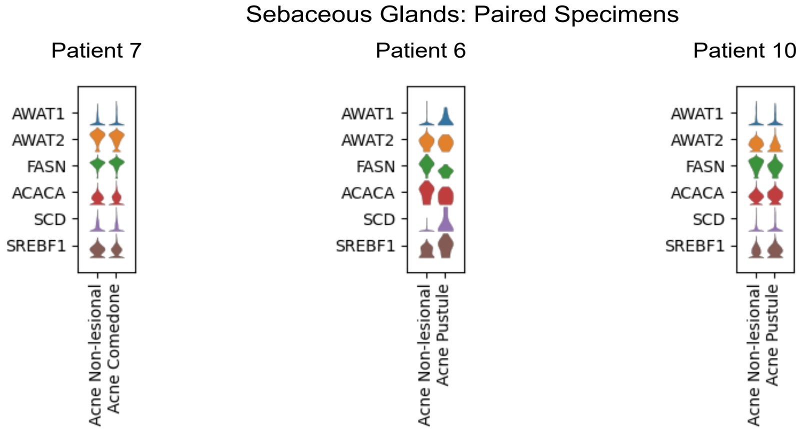
Sebaceous Glands: Paired Specimens
Patient 7
Patient 6
Patient 10
AWAT1
AWAT2
FASN
ACACA
SCD
SREBF1
Acne Non-lesional
Acne Comedone
Acne Pustule

B

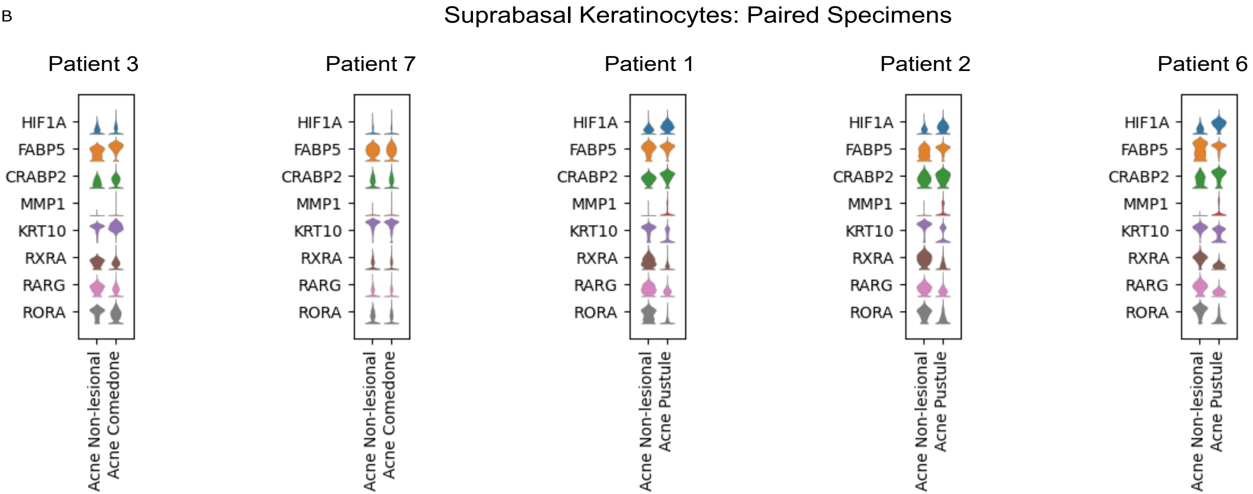
Suprabasal Keratinocytes: Paired Specimens
Patient 3
Patient 7
Patient 1
Patient 2
Patient 6
HIF1A
FABP5
CRABP2
MMP1
KRT10
RXRA
RARG
RORA
Acne Non-lesional
Acne Comedone
Acne Pustule

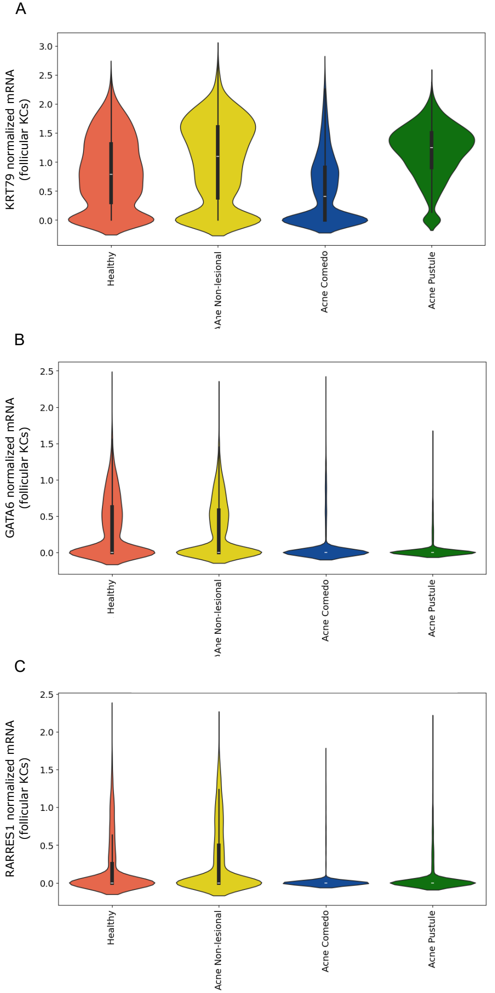
A
KRT79 normalized mRNA
(follicular KCs)
3.0
2.5
2.0
1.5
1.0
0.5
0.0
Healthy
Ane Non-lesional
Acne Comedo
Acne Pustule
B
GATA6 normalized mRNA
(follicular KCs)
2.5
2.0
1.5
1.0
0.5
0.0
Healthy
Ane Non-lesional
Acne Comedo
Acne Pustule
C
RARRES1 normalized mRNA
(follicular KCs)
2.5
2.0
1.5
1.0
0.5
0.0
Healthy
Acne Non-lesional
Acne Comedo
Acne Pustule

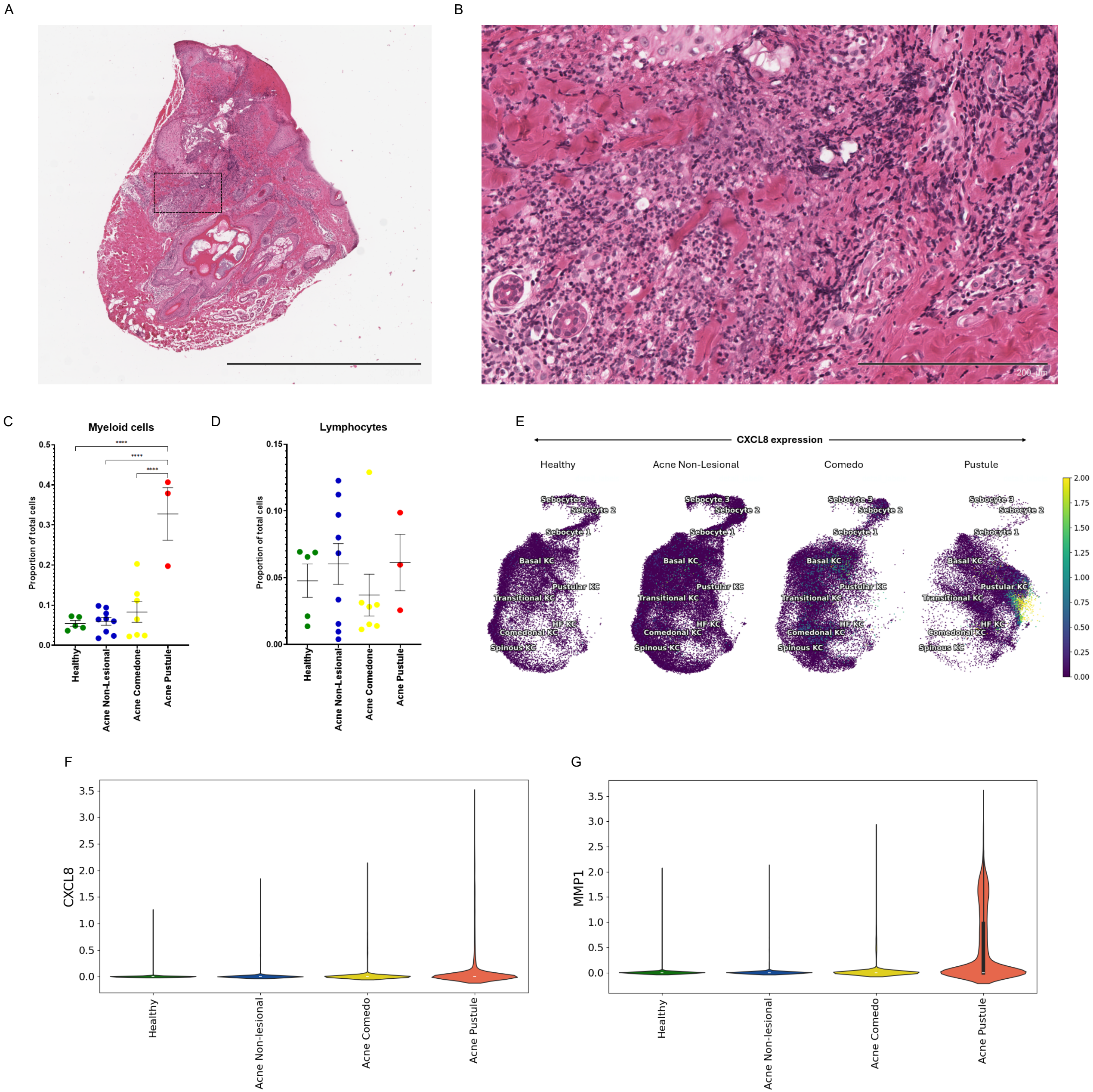

A
B
200 µm
C
Myeloid cells
Proportion of total cells
****
Healthy
Acne Non-Lesional
Acne Comedone
Acne Pustule
D
Lymphocytes
Proportion of total cells
Healthy
Acne Non-Lesional
Acne Comedone
Acne Pustule
E
CXCL8 expression
Healthy
Acne Non-Lesional
Comedo
Pustule
Sebocyte 3
Sebocyte 2
Sebocyte 1
Basal KC
Pustular KC
Transitional KC
HF KC
Comedonal KC
Spinous KC
F
CXCL8
Healthy
Acne Non-lesional
Acne Comedo
Acne Pustule
G
MMP1
Healthy
Acne Non-lesional
Acne Comedo
Acne Pustule

Table S1: Donor features

Patient #	Sex	Age	Race	Type	Biopsy Site	Archival	Use
1	F	31	NR	Comedone	Left superior preauricular	Y	IHC
2	M	20	NR	Non-lesional	Mid back	Y	IHC
3	F	24	NR	Pustule	Left temple	Y	IHC
4	F	22	NR	Non-lesional	Left preauricular	Y	IHC
5	M	18	NR	Pustule	Right forehead	Y	IHC
6	F	26	NR	Pustule	Left temple	Y	IHC
7	M	21	NR	Non-lesional	Left preauricular	Y	Xenium, IHC
7	M	21	NR	Comedone	Right temple	Y	Xenium (x2)
7	M	21	NR	Comedone	Left temple	Y	Xenium, IHC
8	F	32	NR	Non-lesional	Left inferior preauricular	Y	Xenium
9	M	24	NR	Comedone	Right temple	Y	IHC
9	M	24	NR	Pustule	Left preauricular	Y	IHC
9	M	24	NR	Non-lesional	Left inferior preauricular	Y	Xenium, IHC
10	M	18	W	Non-lesional	Right preauricular	N	Xenium (x2), IHC
10	M	18	W	Pustule	R cheek	N	Xenium, IHC
11	M	19	W	Non-lesional	Right preauricular	N	Xenium
11	M	19	W	Pustule	Back	N	Xenium
12	F	33	B	Non-lesional	Back	N	Xenium (x2), IHC
12	F	33	B	Pustule	Back	N	Xenium, IHC
12	F	33	B	Comedone	Face	N	Xenium, IHC
13	F	21	W	Non-lesional	Face	N	Xenium
13	F	21	W	Comedone	Face	N	Xenium
13	F	21	W	Comedone	Back	N	Xenium (x2), IHC
14	F	47	W	Healthy	Left preauricular	N	Xenium, IHC
15	F	27	W	Healthy	Right preauricular	N	Xenium (x2), IHC
16	M	48	B	Healthy	Right preauricular	N	Xenium, IHC
17	M	49	W	Healthy	Left preauricular	N	Xenium, IHC

***: NR, not reported**

Table S2: Custom Xenium panel

Gene	Ensembl ID	Probesets
ACACA	ENSG00000278540	8
AHR	ENSG00000106546	8
AKT1	ENSG00000142208	8
AR	ENSG00000169083	8
AREG	ENSG00000109321	8
ATG7	ENSG00000197548	8
AWAT1	ENSG00000204195	8
AWAT2	ENSG00000147160	8
CAMP	ENSG00000164047	5
CASP1	ENSG00000137752	8
CD163	ENSG00000177575	8
CD1D	ENSG00000158473	8
CD3D	ENSG00000167286	5
CD4	ENSG00000010610	8
CD68	ENSG00000129226	6
CD8A	ENSG00000153563	8
CIDEA	ENSG00000176194	6
COL1A1	ENSG00000108821	8
CRABP1	ENSG00000166426	4
CRABP2	ENSG00000143320	7
CXCL8	ENSG00000169429	8
CYP11A1	ENSG00000140459	8
CYP17A1	ENSG00000148795	8
CYP19A1	ENSG00000137869	8
DGAT1	ENSG00000185000	8
DHCR7	ENSG00000172893	8
DNASE2	ENSG00000105612	5
EGFR	ENSG00000146648	8
ELOVL4	ENSG00000118402	8
FA2H	ENSG00000103089	8
FAAH	ENSG00000117480	8
FABP5	ENSG00000164687	6
FASN	ENSG00000169710	8
FCGR3A	ENSG00000203747	8
FGF10	ENSG00000070193	8
FGF7	ENSG00000140285	8
FGFR2	ENSG00000066468	8
FOXO1	ENSG00000150907	8
FOXO3	ENSG00000118689	8
FOXP3	ENSG00000049768	7

GATA6	ENSG00000141448	8
HIF1A	ENSG00000100644	8
HSD17B2	ENSG00000086696	8
HSD3B1	ENSG00000203857	8
IFNG	ENSG00000111537	8
IGF1	ENSG00000017427	8
IGF1R	ENSG00000140443	8
IGFBP2	ENSG00000115457	3
IGFBP4	ENSG00000141753	6
IL10	ENSG00000136634	8
IL17A	ENSG00000112115	8
IL1B	ENSG00000125538	8
IL6	ENSG00000136244	8
ITGAM	ENSG00000169896	8
KRT10	ENSG00000186395	3
KRT14	ENSG00000186847	3
KRT5	ENSG00000186081	8
KRT7	ENSG00000135480	5
KRT79	ENSG00000185640	8
LCN2	ENSG00000148346	6
LRIG1	ENSG00000144749	8
MAP1LC3B	ENSG00000140941	8
MKI67	ENSG00000148773	8
MMP1	ENSG00000196611	8
MMP3	ENSG00000149968	8
MMP9	ENSG00000100985	6
MTOR	ENSG00000198793	8
MYC	ENSG00000136997	8
NCAM1	ENSG00000149294	8
NLRP3	ENSG00000162711	8
NR1H3	ENSG00000025434	8
NRG4	ENSG00000169752	8
PAPPA	ENSG00000182752	8
PDGFRA	ENSG00000134853	8
PECAM1	ENSG00000261371	8
PPARG	ENSG00000132170	8
PRDM1	ENSG00000057657	8
PSPH	ENSG00000146733	8
RARA	ENSG00000131759	7
RARG	ENSG00000172819	8
RARRES1	ENSG00000118849	8
RASD1	ENSG00000108551	3
RORA	ENSG00000069667	8

RXRA	ENSG00000186350	8
SCD	ENSG00000099194	8
SGPL1	ENSG00000166224	8
SGPP2	ENSG00000163082	8
SPHK2	ENSG00000063176	7
SRD5A1	ENSG00000145545	8
SRD5A2	ENSG00000277893	8
SREBF1	ENSG00000072310	8
TGFB1	ENSG00000105329	7
TLR2	ENSG00000137462	8
TNF	ENSG00000232810	8
TP53	ENSG00000141510	8
TP63	ENSG00000073282	8
TSLP	ENSG00000145777	8
VDR	ENSG00000111424	8
VIM	ENSG00000026025	8
ZBTB16	ENSG00000109906	8

Table S3: Transcription factor binding sites in FABP5 promoter region

Chromosome	Start	End	TF	ID	Position*
chr8	81280037	81280052	FOXH1	ms.FOXH1_HUMAN.48961.v1	499
chr8	81280045	81280145	EP300	ms.EP300_HUMAN.1436299.v1	491
chr8	81280051	81280127	HNF4A	ms.HNF4A_HUMAN.288525.v1	485
chr8	81280054	81280144	CREB1	ms.CREB1_HUMAN.366152.v1	482
chr8	81280070	81280178	SMARCA4	ms.SMCA4_HUMAN.1026051.v1	466
chr8	81280071	81280163	INO80	ms.INO80_HUMAN.97883.v1	465
chr8	81280072	81280140	CUX1	ms.CUX1_HUMAN.8603.v1	464
chr8	81280075	81280175	PAX3	ms.PAX3_HUMAN.49074.v1	461
chr8	81280085	81280156	FOXA1	ms.FOXA1_HUMAN.2177005.v1	451
chr8	81280088	81280162	KAT2B	ms.KAT2B_HUMAN.14931.v1	448
chr8	81280099	81280214	MBD4	ms.MBD4_HUMAN.991086.v1	437
chr8	81280101	81280129	FOXA2	ms.FOXA2_HUMAN.1679287.v1	435
chr8	81280103	81280191	GATAD2A	ms.P66A_HUMAN.276761.v1	433
chr8	81280104	81280208	AR	ms.ANDR_HUMAN.2581248.v1	432
chr8	81280106	81280190	TBP	ms.TBP_HUMAN.260766.v1	430
chr8	81280108	81280137	PDX1	ms.PDX1_HUMAN.143061.v1	428
chr8	81280109	81280195	ONECUT2	ms.ONEC2_HUMAN.417150.v1	427
chr8	81280112	81280206	E2F1	ms.E2F1_HUMAN.299910.v1	424
chr8	81280112	81280206	KDM1A	ms.KDM1A_HUMAN.458680.v1	424
chr8	81280117	81280201	CTCF	ms.CTCF_HUMAN.2220455.v1	419
chr8	81280119	81280201	ETV5	ms.ETV5_HUMAN.67948.v1	417

chr8	81280120	81280224	BRD4	ms.BRD4_HUMAN.2530698.v1	416
chr8	81280120	81280178	RARA	ms.RARA_HUMAN.209768.v1	416
chr8	81280128	81280224	SP1	ms.SP1_HUMAN.333909.v1	408
chr8	81280143	81280233	FLI1	ms.FLI1_HUMAN.626296.v1	393
chr8	81280145	81280237	MCM7	ms.MCM7_HUMAN.188019.v1	391
chr8	81280155	81280241	HNF4A	ms.HNF4A_HUMAN.288526.v1	381
chr8	81280156	81280242	SPIB	ms.SPIB_HUMAN.40573.v1	380
chr8	81280158	81280258	HOXC8	ms.HXC8_HUMAN.4567.v1	378
chr8	81280159	81280213	BRD3	ms.BRD3_HUMAN.150937.v1	377
chr8	81280165	81280249	CDX2	ms.CDX2_HUMAN.267626.v1	371
chr8	81280165	81280256	RARA	ms.RARA_HUMAN.209769.v1	371
chr8	81280170	81280268	PPARG	ms.PPARG_HUMAN.835199.v1	366
chr8	81280170	81280254	ERG	ms.ERG_HUMAN.802439.v1	366
chr8	81280171	81280269	MAZ	ms.MAZ_HUMAN.211826.v1	365
chr8	81280178	81280272	RUNX1	ms.RUNX1_HUMAN.978883.v1	358
chr8	81280192	81280290	ESR1	ms.ESR1_HUMAN.3831196.v1	344
chr8	81280194	81280278	SMAD4	ms.SMAD4_HUMAN.117484.v1	342
chr8	81280207	81280241	ZEB1	ms.ZEB1_HUMAN.179889.v1	329
chr8	81280208	81280266	NR2F6	ms.NR2F6_HUMAN.73422.v1	328
chr8	81280210	81280310	TRPS1	ms.TRPS1_HUMAN.1042699.v1	326
chr8	81280211	81280323	SMAD2	ms.SMAD2_HUMAN.166972.v1	325
chr8	81280211	81280311	JUNB	ms.JUNB_HUMAN.234913.v1	325
chr8	81280214	81280312	KMT2B	ms.KMT2B_HUMAN.245291.v1	322
chr8	81280218	81280322	KMT2A	ms.KMT2A_HUMAN.617547.v1	318
chr8	81280223	81280287	TFAP4	ms.TFAP4_HUMAN.201730.v1	313
chr8	81280225	81280325	KDM4A	ms.KDM4A_HUMAN.63859.v1	311
chr8	81280226	81280326	POU5F1	ms.PO5F1_HUMAN.447852.v1	310
chr8	81280227	81280327	SSU72	ms.SSU72_HUMAN.48048.v1	309
chr8	81280228	81280312	NFATC1	ms.NFAC1_HUMAN.74187.v1	308
chr8	81280229	81280313	FOXA2	ms.FOXA2_HUMAN.1679288.v1	307
chr8	81280230	81280314	TAF15	ms.RBP56_HUMAN.80642.v1	306
chr8	81280231	81280251	AGO1	ms.AGO1_HUMAN.101665.v1	305
chr8	81280232	81280318	INTS11	ms.INT11_HUMAN.337204.v1	304
chr8	81280234	81280310	SMARCA4	ms.SMCA4_HUMAN.1026052.v1	302
chr8	81280240	81280310	NR2F2	ms.COT2_HUMAN.311841.v1	296
chr8	81280241	81280317	ELF1	ms.ELF1_HUMAN.231029.v1	295
chr8	81280245	81280339	RARA	ms.RARA_HUMAN.209770.v1	291
chr8	81280247	81280335	TAF1	ms.TAF1_HUMAN.154277.v1	289
chr8	81280255	81280347	SIRT6	ms.SIR6_HUMAN.147332.v1	281
chr8	81280258	81280344	OGG1	ms.OGG1_HUMAN.102636.v1	278
chr8	81280262	81280358	SP1	ms.SP1_HUMAN.333910.v1	274
chr8	81280263	81280333	FOS	ms.FOS_HUMAN.399094.v1	273
chr8	81280265	81280363	ARID4B	ms.ARI4B_HUMAN.48490.v1	271

chr8	81280267	81280367	GABPB1	ms.GABP1_HUMAN.35118.v1	269
chr8	81280272	81280304	MYC	ms.MYC_HUMAN.1028846.v1	264
chr8	81280277	81280365	JUN	ms.JUN_HUMAN.623530.v1	259
chr8	81280278	81280356	KDM4C	ms.KDM4C_HUMAN.21528.v1	258
chr8	81280280	81280368	KDM2B	ms.KDM2B_HUMAN.111931.v1	256
chr8	81280281	81280347	KLF11	ms.KLF11_HUMAN.39422.v1	255
chr8	81280281	81280387	REST	ms.REST_HUMAN.244390.v1	255
chr8	81280282	81280360	NFYA	ms.NFYA_HUMAN.232784.v1	254
chr8	81280283	81280383	HOMEZ	ms.HOMEZ_HUMAN.42905.v1	253
chr8	81280283	81280383	ZHX2	ms.ZHX2_HUMAN.51199.v1	253
chr8	81280286	81280384	EP300	ms.EP300_HUMAN.1436300.v1	250
chr8	81280286	81280389	E2F8	ms.E2F8_HUMAN.278331.v1	250
chr8	81280289	81280343	BHLHE40	ms.BHE40_HUMAN.105945.v1	247
chr8	81280289	81280387	PHF8	ms.PHF8_HUMAN.68002.v1	247
chr8	81280289	81280312	HIF1A	ms.HIF1A_HUMAN.343929.v1	247
chr8	81280289	81280363	TCF7L1	ms.TF7L1_HUMAN.49073.v1	247
chr8	81280289	81280357	MAX	ms.MAX_HUMAN.428810.v1	247
chr8	81280291	81280377	LDB1	ms.LDB1_HUMAN.137157.v1	245
chr8	81280291	81280359	E2F6	ms.E2F6_HUMAN.87387.v1	245
chr8	81280292	81280322	ETS1	ms.ETS1_HUMAN.569632.v1	244
chr8	81280293	81280371	CHD8	ms.CHD8_HUMAN.112448.v1	243
chr8	81280296	81280388	HIRA	ms.HIRA_HUMAN.129915.v1	240
chr8	81280296	81280394	MECOM	ms.EVI1_HUMAN.107862.v1	240
chr8	81280296	81280386	STAT5B	ms.STA5B_HUMAN.321138.v1	240
chr8	81280297	81280381	SMAD2	ms.SMAD2_HUMAN.166973.v1	239
chr8	81280297	81280381	ZFX	ms.ZFX_HUMAN.115948.v1	239
chr8	81280298	81280394	GATA6	ms.GATA6_HUMAN.235454.v1	238
chr8	81280301	81280401	CDK9	ms.CDK9_HUMAN.334467.v1	235
chr8	81280303	81280323	SRSF4	ms.SRSF4_HUMAN.24728.v1	233
chr8	81280303	81280397	GATA2	ms.GATA2_HUMAN.1083967.v1	233
chr8	81280305	81280395	REL	ms.REL_HUMAN.37541.v1	231
chr8	81280307	81280391	ZEB1	ms.ZEB1_HUMAN.179890.v1	229
chr8	81280307	81280395	KLF4	ms.KLF4_HUMAN.268299.v1	229
chr8	81280309	81280385	ELF1	ms.ELF1_HUMAN.231030.v1	227
chr8	81280312	81280378	SKI	ms.SKI_HUMAN.52596.v1	224
chr8	81280314	81280398	CBX1	ms.CBX1_HUMAN.23418.v1	222
chr8	81280314	81280360	HIC1	ms.HIC1_HUMAN.23468.v1	222
chr8	81280315	81280378	SREBF2	ms.SRBP2_HUMAN.114113.v1	221
chr8	81280316	81280336	HNRNPL	ms.HNRPL_HUMAN.53958.v1	220
chr8	81280318	81280426	SMARCA4	ms.SMCA4_HUMAN.1026053.v1	218
chr8	81280318	81280404	RUNX1	ms.RUNX1_HUMAN.978884.v1	218
chr8	81280318	81280378	TP53	ms.P53_HUMAN.676579.v1	218
chr8	81280318	81280392	TRIM28	ms.TIF1B_HUMAN.335510.v1	218

chr8	81280319	81280347	TP63	ms.P63_HUMAN.498825.v1	217
chr8	81280320	81280344	LARP7	ms.LARP7_HUMAN.44823.v1	216
chr8	81280321	81280349	RORC	ms.RORG_HUMAN.65664.v1	215
chr8	81280322	81280394	GRHL3	ms.GRHL3_HUMAN.105321.v1	214
chr8	81280325	81280391	TP73	ms.P73_HUMAN.31353.v1	211
chr8	81280327	81280421	CEBPA	ms.CEBPA_HUMAN.479001.v1	209
chr8	81280328	81280422	MYCN	ms.MYCN_HUMAN.645032.v1	208
chr8	81280329	81280421	SRSF7	ms.SRSF7_HUMAN.48016.v1	207
chr8	81280330	81280437	HDAC2	ms.HDAC2_HUMAN.468009.v1	206
chr8	81280331	81280415	SMAD4	ms.SMAD4_HUMAN.117485.v1	205
chr8	81280332	81280418	TFAP2C	ms.AP2C_HUMAN.283195.v1	204
chr8	81280333	81280413	ETS1	ms.ETS1_HUMAN.569633.v1	203
chr8	81280334	81280390	CNOT3	ms.CNOT3_HUMAN.120361.v1	202
chr8	81280335	81280392	AR	ms.ANDR_HUMAN.2581249.v1	201
chr8	81280337	81280437	ETV2	ms.ETV2_HUMAN.49458.v1	199
chr8	81280340	81280397	FLI1	ms.FLI1_HUMAN.626297.v1	196
chr8	81280340	81280414	SP140	ms.SP140_HUMAN.129666.v1	196
chr8	81280340	81280428	SSRP1	ms.SSRP1_HUMAN.483183.v1	196
chr8	81280341	81280435	NR3C1	ms.GCR_HUMAN.791569.v1	195
chr8	81280344	81280426	NR2F1	ms.COT1_HUMAN.84445.v1	192
chr8	81280345	81280443	SMARCC1	ms.SMRC1_HUMAN.594313.v1	191
chr8	81280346	81280403	EBF1	ms.COE1_HUMAN.116969.v1	190
chr8	81280349	81280387	RAD21	ms.RAD21_HUMAN.1007981.v1	187
chr8	81280349	81280453	MLLT1	ms.ENL_HUMAN.66259.v1	187
chr8	81280350	81280414	RBM39	ms.RBM39_HUMAN.75449.v1	186
chr8	81280350	81280422	RXRA	ms.RXRA_HUMAN.284117.v1	186
chr8	81280352	81280412	CEBPB	ms.CEBPB_HUMAN.1206235.v1	184
chr8	81280352	81280420	MYC	ms.MYC_HUMAN.1028847.v1	184
chr8	81280352	81280440	KLF5	ms.KLF5_HUMAN.126283.v1	184
chr8	81280353	81280436	VDR	ms.VDR_HUMAN.158998.v1	183
chr8	81280358	81280378	HEXIM1	ms.HEXI1_HUMAN.96133.v1	178
chr8	81280359	81280439	RELA	ms.TF65_HUMAN.1294602.v1	177
chr8	81280360	81280419	E2F1	ms.E2F1_HUMAN.299911.v1	176
chr8	81280361	81280405	PTBP1	ms.PTBP1_HUMAN.60264.v1	175
chr8	81280361	81280451	ZBTB7A	ms.ZBT7A_HUMAN.281770.v1	175
chr8	81280362	81280462	RNF2	ms.RING2_HUMAN.288098.v1	174
chr8	81280363	81280435	APOBEC3B	ms.ABC3B_HUMAN.25798.v1	173
chr8	81280365	81280457	TRIM28	ms.TIF1B_HUMAN.335511.v1	171
chr8	81280366	81280458	IRF4	ms.IRF4_HUMAN.174925.v1	170
chr8	81280368	81280388	AGO2	ms.AGO2_HUMAN.101461.v1	168
chr8	81280368	81280408	GTF2F1	ms.T2FA_HUMAN.69325.v1	168
chr8	81280368	81280446	RELB	ms.RELB_HUMAN.116575.v1	168
chr8	81280372	81280458	NFYA	ms.NFYA_HUMAN.232785.v1	164

chr8	81280377	81280461	ERG	ms.ERG_HUMAN.802440.v1	159
chr8	81280377	81280449	SP5	ms.SP5_HUMAN.175037.v1	159
chr8	81280377	81280465	TRIM24	ms.TIF1A_HUMAN.124990.v1	159
chr8	81280378	81280460	JUND	ms.JUND_HUMAN.256948.v1	158
chr8	81280378	81280478	HNRNPC	ms.HNRPC_HUMAN.19137.v1	158
chr8	81280378	81280428	USF1	ms.USF1_HUMAN.130582.v1	158
chr8	81280379	81280456	SP1	ms.SP1_HUMAN.333911.v1	157
chr8	81280383	81280473	POU5F1	ms.PO5F1_HUMAN.447853.v1	153
chr8	81280384	81280446	E2F6	ms.E2F6_HUMAN.87388.v1	152
chr8	81280385	81280479	TAF1	ms.TAF1_HUMAN.154278.v1	151
chr8	81280386	81280466	CDK9	ms.CDK9_HUMAN.334468.v1	150
chr8	81280387	81280474	NANOG	ms.NANOG_HUMAN.174582.v1	149
chr8	81280389	81280477	ARID2	ms.ARID2_HUMAN.128902.v1	147
chr8	81280389	81280475	PRDM14	ms.PRD14_HUMAN.20973.v1	147
chr8	81280390	81280476	KLF4	ms.KLF4_HUMAN.268300.v1	146
chr8	81280390	81280480	TRPS1	ms.TRPS1_HUMAN.1042700.v1	146
chr8	81280392	81280412	XRCC5	ms.XRCC5_HUMAN.183620.v1	144
chr8	81280394	81280452	SRF	ms.SRF_HUMAN.118617.v1	142
chr8	81280394	81280440	CTCF	ms.CTCF_HUMAN.2220456.v1	142
chr8	81280395	81280489	ZNF143	ms.ZN143_HUMAN.340368.v1	141
chr8	81280395	81280481	ARNT	ms.ARNT_HUMAN.228285.v1	141
chr8	81280396	81280424	HNRNPK	ms.HNRPK_HUMAN.75734.v1	140
chr8	81280396	81280472	ELF1	ms.ELF1_HUMAN.231031.v1	140
chr8	81280398	81280490	BRD9	ms.BRD9_HUMAN.265234.v1	138
chr8	81280400	81280504	SMC1A	ms.SMC1A_HUMAN.162050.v1	136
chr8	81280404	81280437	TFAP2A	ms.AP2A_HUMAN.163124.v1	132
chr8	81280405	81280503	MECOM	ms.EVI1_HUMAN.107863.v1	131
chr8	81280406	81280438	RBM22	ms.RBM22_HUMAN.143658.v1	130
chr8	81280407	81280483	SP2	ms.SP2_HUMAN.46779.v1	129
chr8	81280407	81280497	KDM2B	ms.KDM2B_HUMAN.111932.v1	129
chr8	81280410	81280518	BRD4	ms.BRD4_HUMAN.2530699.v1	126
chr8	81280410	81280498	STAT5B	ms.STA5B_HUMAN.321139.v1	126
chr8	81280410	81280492	STAT1	ms.STAT1_HUMAN.416138.v1	126
chr8	81280411	81280483	NFKB2	ms.NFKB2_HUMAN.96417.v1	125
chr8	81280412	81280518	BRD2	ms.BRD2_HUMAN.379897.v1	124
chr8	81280412	81280494	STAG1	ms.STAG1_HUMAN.101671.v1	124
chr8	81280412	81280496	TBX2	ms.TBX2_HUMAN.37594.v1	124
chr8	81280414	81280496	BCL6	ms.BCL6_HUMAN.154252.v1	122
chr8	81280416	81280436	HNRNPL	ms.HNRPL_HUMAN.53959.v1	120
chr8	81280418	81280468	CTCFL	ms.CTCFL_HUMAN.99700.v1	118
chr8	81280424	81280518	E2F8	ms.E2F8_HUMAN.278332.v1	112
chr8	81280424	81280522	KMT2B	ms.KMT2B_HUMAN.245292.v1	112
chr8	81280424	81280537	FLI1	ms.FLI1_HUMAN.626298.v1	112

chr8	81280424	81280488	NCAPH2	ms.CNDH2_HUMAN.36534.v1	112
chr8	81280424	81280504	STAT3	ms.STAT3_HUMAN.337890.v1	112
chr8	81280425	81280531	REST	ms.REST_HUMAN.244391.v1	111
chr8	81280425	81280525	HMGN3	ms.HMGN3_HUMAN.11659.v1	111
chr8	81280428	81280534	NFYA	ms.NFYA_HUMAN.232786.v1	108
chr8	81280429	81280497	RUNX2	ms.RUNX2_HUMAN.173723.v1	107
chr8	81280433	81280493	RARA	ms.RARA_HUMAN.209771.v1	103
chr8	81280436	81280528	CDK7	ms.CDK7_HUMAN.41485.v1	100
chr8	81280436	81280512	GATA2	ms.GATA2_HUMAN.1083968.v1	100

*: Position indicates the number of base pairs before the FABP5 transcription start site.

Table S4: Antibodies

**Antibodies**		
Rabbit anti-c-Fos	Cell Signaling	Cat # 31254T
Rabbit anti-FABP5	Cell Signaling	Cat # 39926S
Rabbit anti-Ki67	Cell Signaling	Cat # 12202S
Rabbit anti-KRT5	BioLegend	Cat # 905503
Rabbit anti-KRT10	Covance	Cat # PRB-159P
Chicken anti-KRT14	BioLegend	Cat # 906004
Goat anti-KRT79	Santa Cruz	Cat # sc-243156
Rat anti-Ly6G	BioLegend	Cat # 127601
Rabbit anti-PPARG	Cell Signaling	Cat # 2443S
