# Supplementary material for: Spatial transcriptomics identifies differentiation, lipid metabolism, and retinoid pathway alterations in acne vulgaris
Source: JCI Insight. 2026 Feb 9;11(3):e198021. doi: 10.1172/jci.insight.198021 (PMC12892907; doi:10.1172/jci.insight.198021)

Figure 6G: Final Image

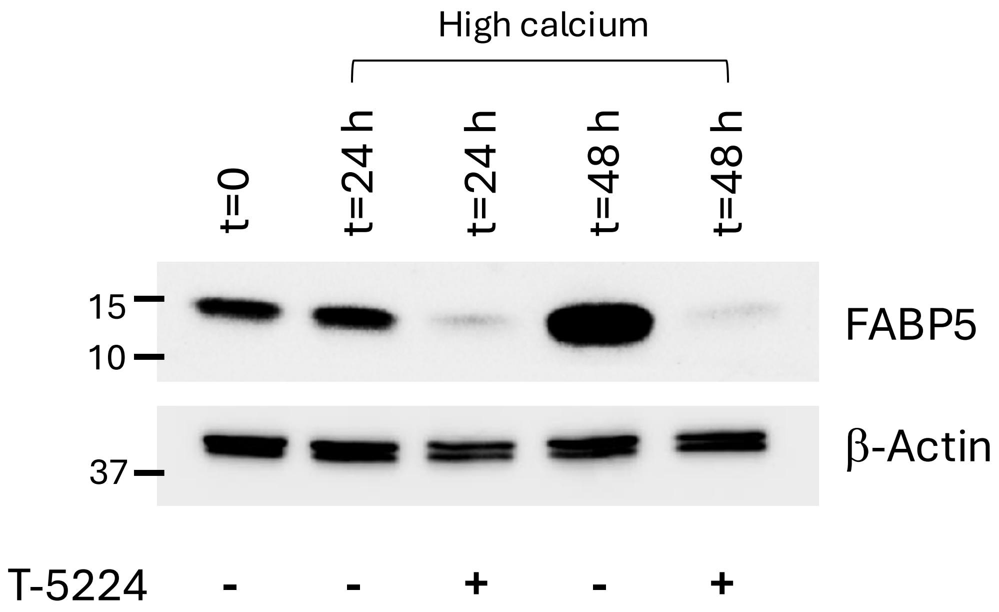

WB (4-20% gel, transfer: 100 V 70 min)

FABP5 Cell signaling (1:1000) (10% Milk blocking, 5% milk for 1st Ab)
Rabbit-HRP (1:3000)
Standard ECL

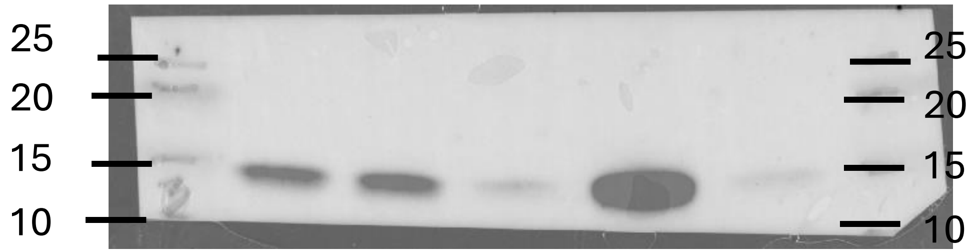

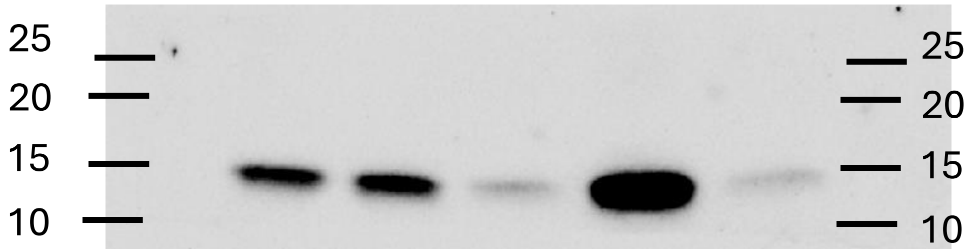

WB (4-20% gel, transfer: 100 V 70 min)

B-Actin Cell signaling (1:10K) (10% Milk blocking, 5% milk for 1st Ab)
Standard ECL

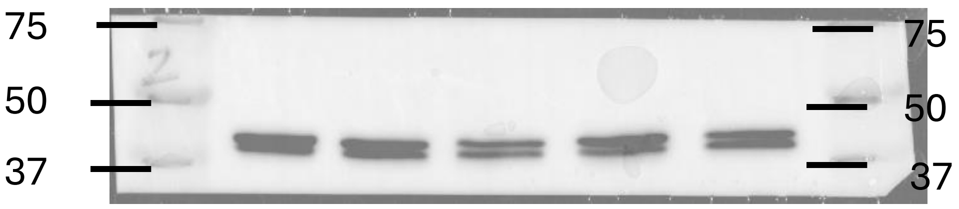

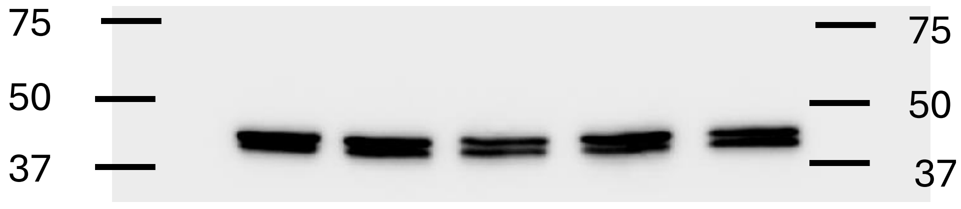

Supplement: Unedited blot and gel images [file jciinsight-11-198021-s116.pdf]
